# 4-Benzyloxylonchocarpin and Muracatanes A-C from *Ranunculus muricatus* L. and Their Biological Effects

**DOI:** 10.3390/biom10111562

**Published:** 2020-11-17

**Authors:** Hidayat Hussain, Iftikhar Ali, Daijie Wang, Nilufar Z. Mamadalieva, Wahid Hussain, René Csuk, Anne Loesche, Lucie Fischer, Dan Staerk, Syariful Anam, Mashail N. AlZain, Maria Mushtaq, Zaheer Ul-Haq, Riaz Ullah, Omar M. Noman, Ghulam Abbas, Ivan R. Green

**Affiliations:** 1 Department of Bioorganic Chemistry, Leibniz Institute of Plant Biochemistry, Weinberg 3, D-06120 Halle (Salle), Germany; Nilufar.Mamadalieva@ipb-halle.de; 2 School of Pharmaceutical Sciences and Key Laboratory for Applied Technology of Sophisticated Analytical Instruments of Shandong Province, Shandong Analysis and Test Center, Qilu University of Technology (Shandong Academy of Sciences), Jinan 250014, China; iftikhar.ali@kiu.edu.pk (I.A.); wangdaijie@qlu.edu.cn (D.W.); 3 Department of Chemistry, Karakoram International University, Gilgit 15100, Pakistan; 4 Department of Botany, Government Post Graduate College Parachinar, Parachinar 26300, District Kurram, Pakistan; wahidhussain@uop.edu.pk; 5 Organic Chemistry, Martin-Luther University Halle-Wittenberg, Kurt-Mothes-Str. 2, D-06120 Halle (Saale), Germany; rene.csuk@chemie.uni-halle.de (R.C.); anne.loesche@web.de (A.L.); lucie.fischer2018@gmx.de (L.F.); 6 Department of Drug Design and Pharmacology, Faculty of Health and Medical Sciences, University of Copenhagen, 1165 Copenhagen, Denmark; ds@sund.ku.dk (D.S.); syarifulanam1@gmail.com (S.A.); 7 Department of Pharmacy, Faculty of Sciences, Tadulako University, Palu 94118, Indonesia; 8 Department of Biology, College of Sciences, Princess Nourah Bint Abdulrahman University, Riyadh 11451, Saudi Arabia; 9 Dr. Panjwani Center for Molecular Medicine and Drug Research, International Center for Chemical and Biological Sciences, University of Karachi, Karachi 75270, Pakistan; mariahasnain@gmail.com (M.M.); zaheer.qasmi@iccs.edu (Z.U.-H.); 10 Department of Pharmacognosy (MAPPRC), College of Pharmacy, King Saud University, P.O. Box 2457, Riyadh 11451, Saudi Arabia; rullah@ksu.edu.sa (R.U.); onoman@ksu.edu.sa (O.M.N.); 11 Department of Biological Sciences and Chemistry, College of Arts and Sciences, University of Nizwa, Nizwa 616, Oman; abbashej@unizwa.edu.om; 12 Department of Chemistry and Polymer Science, University of Stellenbosch, Private Bag X1, Matieland, Stellenbosch 7600, South Africa; irg@sun.ac.za

**Keywords:** *Ranunculus muricatus* L, structure elucidation, acetylcholinesterase, α-glucosidase

## Abstract

*Ranunculus muricatus* L. is a spiny fruit buttercup that is used in various traditional medicinal systems. In the current investigation of *R. muricatus*, the new chalcone 4-benzyloxylonchocarpin (**1**), the new anthraquinone muracatanes A (**2**), the new-to-nature anthraquinone muracatanes B (**3**), and the new naphthalene analog muracatanes C (**4**) were isolated, in addition to the three previously reported compounds, 4-methoxylonchocarpin (**5**), β-sitosterol (**6**), and β-sitosterol β-D-glucopyranoside (**7**). Their structures were elucidated using 1D (^1^H and ^13^C) and 2D (COSY, HSQC, and HMBC) NMR spectroscopy and HR-ESI-MS. Chalcone **1** showed potent acetylcholinesterase inhibitory effects with *K*_i_ of 5.39 µM and *K*_i′_ of 3.54 µM, but none of the isolated compounds showed inhibitory activity towards butyrylcholinesterase. Anthraquinone **3** illustrated α-glucosidase inhibitory effects with IC_50_-values of 164.46 ± 83.04 µM. Compound **5** displayed moderate cytotoxic activity towards ovarian carcinoma (A2780, IC_50_ = 25.4 µM), colorectal adenocarcinoma (HT29, IC_50_ = 20.2 µM), breast cancer (MCF7, IC_50_ = 23.7 µM), and thyroid carcinoma (SW1736, IC_50_ = 26.2 µM) while it was inactive towards pharynx carcinoma (FaDu: IC_50_ > 30 µM).

## 1. Introduction

Natural products (NPs) are considered to be tremendously important sources of inspiration for drug discovery [1,2,3]. More than half of the drugs approved between 1981 and 2014 were derived from natural products, i.e., being unaltered natural compounds or their synthetic derivatives [1]. Moreover, the U.S. Food and Drug Administration (FDA) illustrated that NPs and their analogues constitute over one-third of all FDA-approved new chemical entities [3], and this is in particular true for anticancer drugs and antibiotics [1], which are significantly enriched with natural products or natural product derivatives [1,4,5,6,7]. The *Ranunculus* genus comprises 600 species and *R. muricatus* L. is named so because of its spiny fruit buttercup. This plant is widely present in Asia, Australia, South America, and Europe [8,9]. *R. muricatus* is used in traditional medicine to treat cough, asthma [10], dysentery, jaundice, diarrhea, eczema, urinary infections, leprosy, and ringworm infection [11,12]. *R. muricatus* has also been employed in folk medicine to treat cancer, heart diseases, and dental diseases [13,14,15], and *R. muricatus* extracts have been reported to possess antioxidant [16], antifungal, antibacterial, and cytotoxic effects [17]. *R. muricatus* is furthermore employed to treat tonsillitis diseases in traditional medicine protocols in India [18]. Moreover, other *Ranunculus* species, such as *R. termatus, R. japonicas,* and *R. sceleratus* have found use in a wide range of clinical applications in Chinese Traditional Medicine to treat scrofula, lymphatic tuberculosis, swollen hemorrhoids, malaria, and arthritis [19,20,21]. Various natural products have been reported from *R. muricatus*, which include tannins, saponins, flavonoids, phenols, alkaloids, anthocyanins, cardiac glycosides, phytosterols, carbohydrates, coumarins, and diterpenes [15,22,23].

Alzheimer’s disease (AD) is a complex neurodegenerative disorder illustrated by intellectual reduction, memory loss, and anomalous behavior, and it is evolving as a global health problem in aging populations. Patients with AD show decreased levels of acetylcholine (ACh) and butyrylcholine (BCh), which act as neurotransmitters [24]. Acetylcholinesterase (AChE) and butyrylcholinesterase (BChE) hydrolyse ACh and BCh into acetyl and butyryl, respectively, and choline. Inhibitors of AChE and BChE thus result in both an increased level and increased duration of the neurotransmitter action, which is important for management of AD [25].

α-Glucosidase hydrolyses complex carbohydrates into absorbable monosaccharides, and is therefore an attractive therapeutic target for management of blood glucose of type 2 diabetics. However, currently available α-glucosidase inhibitors are associated with side effect such as gas, bloating and mild diarrhea, and discovery of new natural products with α-glucosidase inhibitory activity and fewer side effects are highly desired [26]. In the current investigation, four new natural products **1–4** along with the known metabolites **5–7** were found in *R. muricatus*, and these compounds were evaluated for AChE, BChE, and α-glucosidase inhibitory activity as well as cytotoxicity.

## 2. Material and Methods

### 2.1. General Experimental Procedures and Chemicals

IR spectra were recorded using a Nicolet-510P spectrophotometer (Thermo Fisher Scientific, Waltham, MA, USA). All NMR experiments were acquired using a Bruker AMX instrument operating at a ^1^H frequency of 400.13 MHz (Bruker Biospin, Karlsruhe, Germany). HR-ESI-MS were obtained on a Thermo Fischer LTQ Orbitrap elite mass spectrometer (Thermo Fisher Scientific, Waltham, MA, USA). Column chromatography was carried out using silica gel (70–230 and 230–400 mesh; Merck, Darmstadt, Germany). A TECAN SpectraFluor Plus (Tecan Life Sciences, Zürich, Switzerland) working in the kinetic mode was used for the enzymatic studies thereby measuring the absorbance at 415 nm. AChE (from *Electrophorus electricus*), 5,5′-dithiobis-(2-nitrobenzoic acid) (DNTB), acetylthiocholine iodide (ATChI), BChE (from equine serum), and butyrylthiocholine iodide (BTChI) were obtained from Merck (Darmstadt, Germany). α-Glucosidase type I (EC 3.2.1.20 from *Saccharomyces cerevisiae*, lyophilized powder), *p*-nitrophenol α-D-glucopyranoside (*p*-NPG), dimethyl sulfoxide (DMSO), sodium dihydrogen phosphate dihydrate, disodium hydrogen phosphate, sodium azide, and acarbose were purchased from Sigma-Aldrich (St. Louis, MO, USA).

### 2.2. Plant Material

Whole plant material of *R. muricatus* was collected from Parachinar, KPK, Pakistan, in July 2015 and identified by Dr. Wahid Hussain (plant taxonomist). The voucher specimen (No. B. Huss.105. GPGC PCR) has been deposited at the herbarium of the Department Botany, Post Graduate College Parachinar, Pakistan.

### 2.3. Extraction and Isolation

A total of 3.4 kg of *R. muricatus* was extracted with 10 L of EtOH at room temperature, and the solvent was removed on a rotary evaporator providing 6.5 g of residue, which was separated by column chromatography (*n*-hexane, *n*-hexane–EtOAc and EtOAc [from 5:95 to 10:90]) to yield fractions F1 to F8). Fraction F3 (120 mg) was re-chromatographed on 25 g of silica gel with a mixture of *n*-hexane–EtOAc (1.8:8.2) as eluent yielding 4-benzyloxylonchocarpin (**1**; 7.9 mg) and 4-methoxylonchocarpin (**5**; 5.3 mg). Fraction, F5 (155 mg) was re-chromatographed on 20 g of silica gel and eluted with *n*-hexane–EtOAc (7.5:2.5 to 5:5) as eluent to afford muracatane A (**2**; 10.4 mg), muracatane B (**3**; 5.6 mg), and β-sitosterol β-D-glucopyranoside (**7**; 11.9 mg). Finally, muracatane A (**4**; 3.2 mg) and β-sitosterol (**6**; 22.3 mg) were obtained from fraction F2 (120 mg) re-chromatographing this fraction on 18 g of silica gel and eluting with *n*-hexane–EtOAc (1.2:8.8).

*4-Benzyloxylonchocarpin* (**1**). Yellow solid; IR (KBr) v_max_: 3310, 1655, 1610, 1420, 1000 cm^−1^; for ^1^H (400 MHz, CDCl_3_) and ^13^C NMR (100 MHz, CDCl_3_) see Table 1; HRESIMS: m/z 413.1711 [M+H]^+^: (calcd for C_27_H_25_O_4_^+^, 413.1747, ΔM 8.8 ppm).

*Muracatane A* (**2**). Yellow solid; IR (KBr) v_max_: 1605, 1415, 1010 cm^−1^; for ^1^H (400 MHz, CDCl_3_) and ^13^C NMR (100 MHz, CDCl_3_) see Table 1; HRESIMS: m/z 413.1235 [M+H]^+^: (calcd for C_22_H_21_O_8_^+^, 413.1231, ΔM −1.0 ppm).

*Muracatane B* (**3**). Yellow solid; IR (KBr) v_max_: 3350, 1610, 1430, 1000 cm^−1^; for ^1^H (400 MHz, DMSO-d_6_) and ^13^C NMR (100 MHz, DMSO-d_6_) see Table 2; HRESIMS: m/z 257.0450 [M+H]^+^: (calcd for C_14_H_9_O_5_^+^, 257.0445, ΔM −1.0 ppm).

*Muracatane C* (**4**). White solid; IR (KBr) v_max_: 1600, 1420, 1000 cm^−1^; for ^1^H (400 MHz, CDCl_3_) and ^13^C NMR (100 MHz, CDCl_3_) see Table 2; HRESIMS: m/z 217.1233 [M+H]: (calcd for C_14_H_17_O_2_^+^, 217.1223, ΔM −4.6 ppm).

### 2.4. Solutions Preparation for AChE and BChE

A solution of tris(hydroxymethyl)-aminomethane (606 mg) in twice-distilled water (100 mL) was adjusted to a pH of 8.0 ± 0.1 with HCl to get a 50 mM Tris-HCl buffer solution. The AChE solution holding 2.005 U/mL was prepared by dissolving the enzyme (271 U/mg, 0.037 mg) in freshly prepared buffer pH 8.0 (5 mL) comprising extra NaN_3_ (0.98 mg), and for the BChE solution with the concentration of 2.040 U/mL: the enzyme (7.54 U/mg, 1.353 mg) was dissolved in freshly prepared buffer pH 8.0 (5 mL) comprising NaN_3_ (0.98 mg). The DTNB solution with the concentration of 3 mM was prepared by dissolving DTNB (23.8 mg) in buffer pH 8.0 (20 mL) and this was followed by the addition of MgCl_2_ (38.0 mg) and NaCl (116.8 mg). The ATChI solution with the concentration of 15 mM was prepared by dissolving ATChI (43.4 mg) in twice-distilled water (10 mL). Furthermore, all solutions were stored in Eppendorf tubes in the refrigerator or freezer. The tested natural products were dissolved in DMSO, galantamine hydrobromide was used as a reference compound was dissolved in twice-distilled water. The final concentrations for the enzymatic assays were obtained by diluting the stock solution with twice-distilled water. No inhibition was detected by residual DMSO (<0.5%).

### 2.5. Cholinesterase Assay

A mixture of the tested natural product solutions (25 µL, 3 different concentrations and one blank), DTNB solution (125 μL) and enzyme solution (25 μL, AChE or BChE) was incubated at 30 °C for 20 min in 96-well microplates followed by the addition of the substrate (25 μL, at 4 different concentrations). The final substrate concentrations were as follows: [ATChI] = 0.9375 mM, 0.625 mM, 0.325 mM, and 0.1875 mM. Moreover, the absorbance (λ = 415 nm) was measured at 30 °C for 30 min at 1 min intervals. All reactions were performed in triplicate with Galantamine hydrobromide as the reference compound. K_i_ and K_i’_ were deduced from Lineweaver–Burk, Dixon, and Cornish–Bowden plots as well as the mode of inhibtion. The relative inhibition was deduced as the quotient of the slopes (compound divided by blank) of the linear ranges. The concentration of each compound was 10 µM while the concentration was ATChI = 0.625 mM. Absorbance data (λ = 415 nm) were recorded at 30 °C for 10 min, and the IC_50_ values were calculated using GraphPad Prism 5 software.

### 2.6. α-Glucosidase Assay

The α-glucosidase assay was applied to pure natural product **3** according to the protocol described by Schmidt and co-workers [27]. In short, the assays were performed in 96-well microplates using a Multiskan FC microplate photometer (Thermo Fisher Scientific, Waltham, MA, USA) controlled by SkanIt 2.5.1 software (Thermo Fisher Scientific, Waltham, MA, USA) with final volumes of 200 µL in each well. A phosphate buffer (100 mM) was prepared by dissolving 2.65 g sodium dihydrogen phosphate dihydrate, 4.70 g disodium hydrogen phosphate, and 0.10 g sodium azide in 500 mL Milli-Q water and adjusting the pH to 7.5. To each well, 10 µL of DMSO sample solution (final well concentration of 10 µM for initial experiments or dilution series with final well concentrations of 395.0, 276.5, 197.5, 98.75, 49.37, 24.69, and 12.34 µM for IC_50_ determination of compound **3** and 2000.0, 1000.0, 500.0, 250.0, 125.0, 62.5, 31.25, 15.62, 7.81 µM for IC_50_ determination of the reference compound, acarbose) was added, followed by 90 µL of a 100 mM phosphate buffer, and 80 µL α-glucosidase enzyme solution (2 U/mL in 100 mM phosphate buffer). The microplate was shaken for 2 min, and incubated for 10 min at 28 °C. Then, 20 µL of *p*-NPG substrate (10 mM, 60.2 mg in 20 mL buffer) was added to each well to initiate the reaction. The enzyme activity (expressed as cleavage rate ΔAU/s) was determined by measuring the absorbance at 405 nm every 30 s for 35 min, and percentage inhibition was calculated according to the equation:

$$\mathrm{Percent}\;\mathrm{inhibition}=\frac{Slope\left(blank\right)-Slope\left(sample\right)}{Slope\left(blank\right)}\times \;100\%$$

where slope_blank_ is the enzyme activity of the wells containing buffer, enzyme, and substrate and slope_sample_ is the enzyme activity in the wells containing buffer, enzyme, substrate, and sample. Dilution series with test solution and the reference compound acarbose were measured in triplicate and used for determination of IC_50_ valuesusing the below four-parameter equation in GraFit version 5.0 (Erithacus Software Limited, Horley, Surrey, RH6 9YJ, UK):

$$f\left(x\right)=min+\;\frac{max-min}{1+{\left(\frac{x}{IC_{50}}\right)}^{slope}}$$

where x is the concentration of the test compound, slope is the Hill slope, and min and max are the minimum and maximum concentrations for the sigmoidal curve.

### 2.7. Sulforhodamine B Assay

The cytotoxicity of the natural products was screened by using the sulforhodamine-B (Kiton Red S, ABCR, Karlsruhe, Germany) in micro culture colorimetric assay using 96-well plates with the seeding of the cells on day 0 applying appropriate cell densities to prevent confluence of the cells during the period of the experiment. On day 1, the cells were treated with six different concentrations (1, 3, 7, 12, 20 and 30 µM); thereby, the final concentration of DMSO was always ≤ 0.5%, generally regarded as non-toxic to the cells. On day 4, the supernatant medium was discarded; the cells were fixed with 10 % trichloroacetic acid. After another day at 4 °C, the cells were washed in a strip washer and dyed with the SRB solution (100 µL, 0.4% in 1% acetic acid) for about 20 min and this was followed by washing of the plates (four times, 1% acetic acid) and air-drying overnight. Furthermore, tris base solution (200 µL, 10 mM) was added to each well and absorbance was measured at λ = 570 nm employing a reader (96 wells, Tecan Spectra, Crailsheim, Germany). The IC_50_ values were averaged from three independent experiments performed each in triplicate calculated from semi logarithmic dose response curves applying a non-linear four-parameter Hills-slope equation (GraphPad Prism5; variables top and bottom were set to 100 and 0, respectively).

### 2.8. Molecular Docking

To investigate the possible mode of inhibition of the proposed compounds **1** and **3**, molecular docking studies were performed with AChE and α-glucosidase, respectively. Molecular docking between a target protein and small molecule is an important tool to understand their interactions. The crystal structure of α-glucosidase was not available and, thus, it was obtained by homology modelling. The homology modeling of α-glucosidase from baker’s yeast was accomplished with SWISS model by following a protocol as mentioned in our previous publication [28], using the protein isomaltase (PDB 3A4A, resolution = 1.3 Å) as a template with identity of 72% and 85% similarity when compared to our target protein. However, the crystal structure of AChE complexed with galantamine (PDB code 4EY6, resolution = 2.4 Å) was retrieved from the Protein Databank. We utilized Homo sapiens AChE structure for docking which shares 62% identity and 52% similarity with the structure AChE utilized for experimental studies. The proteins were prepared by removing water and ligand molecules other than the co-crystallized ligand using Molecular Operating Environment (MOE 2016.0802) [29]. It was followed by protonation using default parameters and energy minimization by AMBER:10EHT force field.

**Figure 1:**
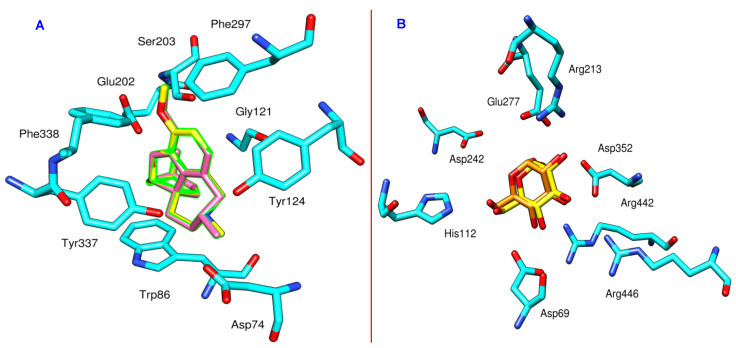
(**A**): Comparison between the docked pose of galantamine derivative (pink) and the co-crystallized ligand (yellow) within AChE (cyan, PDB code: 4EY6); (**B**) comparison between the docked pose of *α*-D-glucose (orange) and the co-crystallized ligand (yellow) within *α*-glucosidase (cyan, PDB code: 3A4A).

The structures of the hit compounds were drawn by using ChemBioDraw Ultra 14.0. This was followed by preparation with MOE by computing protonation states, assigning partial charges and energy minimization by applying MMFF94X force field. The same procedure was followed to prepare co-crystallized ligands for redocking calculations. In order to validate the reproducibility of Molecular Operating Environment (MOE) software, the cognate ligands were re-docked and the RMSD values between the crystal pose and docked pose were found to be 0.75 and 0.08 Å for isomaltase and AChE, respectively (Figure 1A,B), which validates the predictive ability and reproducibility of MOE. After benchmarking, the selected ligands were docked in the binding pocket and scoring was set at London dG while refinement was done with the GBVI/WSA dG scoring function. The induced fit scheme was used for the refinement of the top 30 poses. To study the interactions, post docking analysis was performed using PLIF module implemented in MOE.

## 3. Results

### 3.1. Structure Elucidation

**Figure 2:**
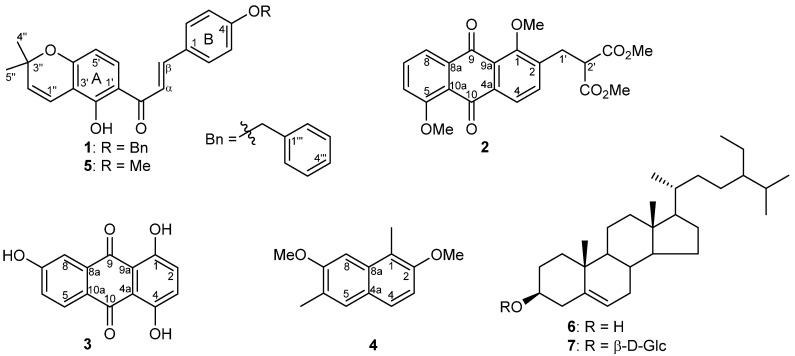
Structures of compounds **1–7** isolated from *R. muricatus*.

**Figure 3:**
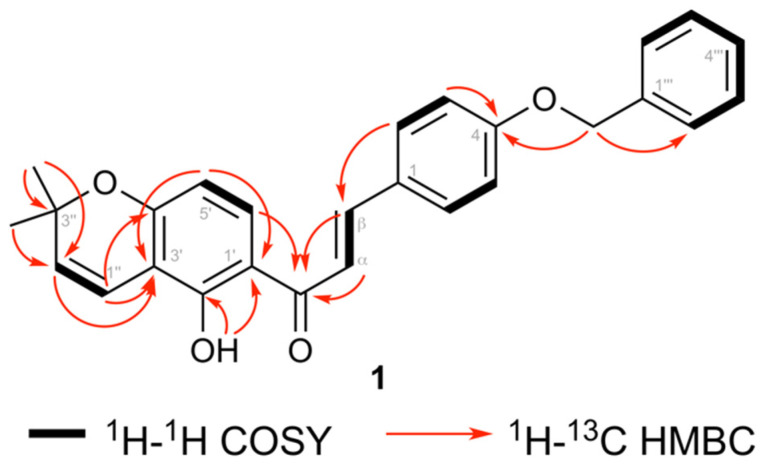
Selected COSY and HMBC correlations for 4-benzyloxylonchocarpin (**1**).

Compound **1** (Figure 2) was obtained as a yellow solid and its molecular formula (C_27_H_24_O_4_) was determined based on HRESIMS. The IR spectrum showed absorption bands for a phenolic OH (3310 cm^−1^), a conjugated ketone (1655 cm^−1^), and aromatic ring systems (1610 cm^−1^). Furthermore, the ^1^H NMR spectrum showed doublet signals at δ 7.45 and 7.86 with a 16.0 Hz coupling constant, which are typical for H-α and H-β of a trans chalcone. This was further confirmed from ^13^C NMR signals for a chalcone at δ 127.8 (C-β), 144.0 (C-α), and 191.9 (conjugated keto). Furthermore, the ^1^H NMR spectrum showed doublets at δ 6.76 (*J =* 10 Hz, H-1′′), and 5.59 (*J* = 10 Hz, H-2′′) along with a 6-proton singlet at δ 1.47, which illustrated the presence of a pyran ring formed via cyclization of a prenyl group with a neighboring hydroxyl group. Furthermore, two doublets at δ 6.37 (1H, *J* = 9.0 Hz) and 7.72 (1H, *J* = 9.0 Hz) was observed for ring A and these protons were assigned to H-5′ and H-6′, respectively, based on their HMBC correlations (Figure 3).

The B-ring substitution at C-4 was confirmed in the ^1^H NMR spectrum by the presence of an AA′BB′ system at δ 7.00 (2H, AA′, H-3 and H-5) and 7.60 (2H, BB′, H-2 and H-6). Moreover, a singlet for a strongly chelated OH group at δ 13.77 and its HMBC correlations to C-1′, C-2′, and C-3′ confirmed this OH to be at C-2′. The position of the prenyl group cyclized into the pyran ring at C-3′ and C-4′ of ring A was confirmed via HMBC correlations from H-1′′ to C-2′, C-3′, and C-4′, and from H-2′′ to C-3′. The ^1^H NMR spectral data of compound **1** was furthermore similar to 4-hydroxylonchocarpin [30] except for the additional signals for a benzyl group at δ 7.32–7.46 (5H, m, Ph) and 5.12 (2H, s, C*H*_2_Ph). The benzyl group was further confirmed from ^13^C NMR signals at δ 128.4 (C-2′′′ and C-6′′′), 128.6 (C-3′′′ and C-5′′′), 127.8 (C-4′′′), 136.3 (C-1′′′), and 70.1 (*C*H_2_Ph). The position of the benzyl group was established via HMBC correlations from the signal at δ 5.12 (C*H*_2_Ph) to C-4. Previously, benzylated secondary metabolites have been reported from fungi [31]. Based on the spectroscopic evidence, the structure of **1** was established as (*E*)-3-(4-(benzyloxy)phenyl)-1-(5-hydroxy-2,2-dimethyl-2*H*-chromen-6-yl)prop-2-en-1-one.

Muracatane A (**2**) was obtained as a yellow powder. The molecular formula (C_22_H_20_O_8_) was determined via HRESIMS and provided an [M + H]^+^ ion at 413.1235 (calcd for C_22_H_21_O_8_^+^, 413.1231), and a proposed 13 degrees of unsaturation. Furthermore, the ^1^H NMR spectrum illustrates two 1-proton *ortho*-coupled aromatic signals at δ 8.00 (d, *J* = 8.0 Hz, H-4) and 7.60 (d, *J* = 8.0 Hz, H-3) along with two methoxy signals at δ 4.04 (3H, s, 5-OMe) and 3.97 (3H, s, 1-OMe). The ^1^H NMR spectrum also illustrates three coupled aromatic 1-proton signals at δ 7.90 (dd, *J* = 8.0, 2.0 Hz), 7.70 (t, *J* = 8.0 Hz), and 7.30 (dd, *J* = 8.0, 2.0 Hz) which are assigned to H-8, H-7 and H-6, respectively. The ^13^C NMR spectrum of **2** showed 16 sp^2^ carbon signals, among which were two oxygenated aromatic carbon signals at δ 159.8 and 158.7 and two signals at δ 182.7, 182.1 for the doubly-conjugated carbonyl carbons of the anthraquinone, and an ester carbonyl at δ 169.0 (with double intensity) which taken together suggested the presence of a substituted dihydroxyanthraquinone skeleton Table 1.

**Figure 4:**
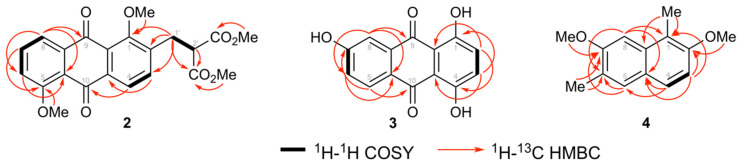
Selected COSY and HMBC correlations for muracatanes A-C (**2**–**4**).

The regiochemistry of substituents on the ring A of the anthraquinone core was determined by COSY and HMBC spectral analysis as shown in Figure 4. COSY correlations from H-8 to H-7 to H-6 together with HMBC correlations from H-8 to C-6, C-10a, and C-9, from H-6 to C-5, C-8, and C-8a, and from H-7 to C-5, C-7, C-8, and C-10a, indicated a 1,2,3-trisubstitued aromatic ring with a methoxy at C-5. Furthermore, ^1^H NMR data showed signals for a dimethyl 2-(methyl)malonate group at δ 3.90 (1H, t, *J* = 8.0 Hz, H-2ꞌ), 3.70 (6H, s, 2 × CO_2_*M*e), and 3.34 (2H,d, *J* = 8.0 Hz, H-1′), which was further confirmed via ^13^C NMR signals at δ 52.6 (CO_2_*M*e), 51.3 (C-2′), and 30.1 (C-1′). The presence of the dimethyl 2-(methyl)malonate group was further confirmed via COSY correlations (Figure 4) and supported by HMBC correlations. The dimethyl 2-(methyl)malonate group positioned at C-2 and the methoxy group positioned at C-1 were confirmed through the following HMBC correlations: H-1′ to C-1, C-2, and C-3; H-3 to C-1′; and OMe-1 (δ 3.97) to C-1. Consequently the structure of muracatane A (**2**) was established as dimethyl 2-((1,5-dimethoxy-9,10-dioxo-9,10-dihydroanthracen-2-yl)methyl)malonate.

Muracatane B (**3**) was isolated as a yellow powder. Moreover, the molecular formula (C_14_H_8_O_5_) was determined via HRESIMS and provided an [M+H]^+^ ion at *m/z* 257.0450 (calcd for C_14_H_9_O_5_^+^, 257.0445) and thus a molecule with a proposed 11 degrees of unsaturation. The ^1^H NMR spectrum possesses two strongly chelated signals for OH groups at δ 12.84 (OH-4) and 12.53 (OH-1) and one non-chelated OH at δ 11.18 (OH-7). Moreover, the ^1^H NMR spectrum showed three coupled aromatic proton signals at δ 7.98 (1H, d, *J* = 8.0 Hz, H-5), 7.40 (1H, d, *J* = 2.0 Hz, H-8), and 7.20 (1H, dd, *J* = 8.0, 2.0 Hz, H-6), corresponding to a 1,2,4-trisubstituted benzene moiety, and a 2-proton signal at δ 7.28 (2H, m, H-2, H-3). Furthermore, the ^13^C NMR spectrum of **3** showed fourteen sp^2^ carbon signals, which included three oxygenated carbon signals at δ 163.6, 156.5, and 156.4. In addition, the ^13^C NMR spectrum showed two downfield signals at δ 186.3 and 185.3 typical for doubly-conjugated carbonyl carbons, which thus suggested the presence of the anthraquinone skeleton. The chemical shift values higher than 185 ppm for the two anthraquinone carbonyl groups confirmed that both these keto groups are hydrogen-bonded to the OH groups [32], as also evidenced by the presence of the two downfield-shifted OH groups in the ^1^H NMR spectrum (δ 12.84 and 12.53) Table 2.

The regiochemistry of the substituents on the A-ring of the anthraquinone core was established via COSY and HMBC spectral analysis as shown in Figure 4. COSY correlations from H-5 to H-6 (*ortho*) to H-8 (*meta*), together with HMBC correlations from H-8 to C-6, C-10a, and C-9, from H-6 to C-5, C-7, C-8, and C-10a, and from H-5 to C-6, C-7, C-10, and C-10a, indicated the 1,2,3-trisubstitued aromatic ring with a hydroxy group at C-7. Furthermore, HMBC correlations from OH-4 (δ 12.84) to C-3, C-4, and C-4a, from OH-1 (δ 12.53) to C-1, C-2, and C-9a, and from H-2/H-3 to C-2, C-3, C-4, C-4a, and C-9a established the position of the chelated OHs at C-1 and C-4 as we well as the regiochemistry of ring C. Consequently, the structure of muracatane B (**3**) was established as 1,4,6-trihydroxyanthracene-9,10-dione. Compound **3** has previously been reported as a synthetic compound [33,34,35], but this is the first report of **3** isolated as a natural product. In order to check that **3** in fact is a true natural product and not an artifact from contamination with commercially available **3**, the plant was freshly extracted in order to crosscheck the presence of the anthraquinone in the crude extract. Compound **3** was detected and isolated again from the second extract of *R. muricatus*, and is thus reported as a new-to-nature compound.

Muracatane C (**4**) was isolated as a light yellow solid. Furthermore, its molecular formula was established to be C_14_H_16_O_2_ by HRESIMS as well as 1D and 2D NMR spectroscopy. The IR spectrum illustrates the presence of a benzene ring (1615 and 1425 cm^−1^). Additionally, the ^1^H NMR spectrum showed two *ortho*-coupled doublets at δ 7.09 (1H, *J* = 8.0 Hz, H-3) and 7.56 (1H, *J* = 8.0 Hz, H-4) along with two aromatic singlets at δ 7.49 (H-5) and 7.08 (H-8). The ^1^H NMR spectrum (data in Table 2) furthermore showed the presence of two methoxy groups (δ_H_ 3.95, δ_C_ 55.1 and δ_H_ 3.91, δ_C_ 56.6) and two methyl groups attached to aromatic ring (δ_H_ 2.50, δ_C_ 16.5, and δ_H_ 2.33, δ_C_ 10.7). Based on these observations, it was suggested that compound **4** has a naphthalene skeleton bearing disubstituted A and B rings [36,37,38,39,40].

^13^C NMR and DEPT) spectral data (Table 2) of **4** showed fourteen carbon signals attributed to four aromatic methine carbon signals, two methoxy and two aromatic methyl signals, and six quaternary carbons. The regiochemistry of one of the methoxy groups and one of the aromatic methyl groups in ring A was determined from HMBC correlations from 7-OMe to C-7, from H-8 to C-6, C-7, and C-8a, from 6-Me to C-5, C-6, and C-7, and from H-5 to C-4a, C-4, C-6, and C-7. HMBC correlations from 1-Me to C-1, C-2, and C-8a, from 2-OMe to C-2, from H-3 to C-1, C-2, C-4, C-4a, and from H-4 to C-2, C-3, and C-4a established the ring B regiochemistry (Figure 4). Consequently, muracatane C (**4**) structure was established to be 2,7-dimethoxy-1,6-dimethylnaphthalene. The three reported secondary metabolites 4-methoxylonchocarpin (**5**) [41], β-sitosterol (**6**) [42], β-sitosterol β-D-glucopyranoside (**7**) [43] were identified by comparing their NMR spectral data with data published in literature.

### 3.2. Biological Evaluation

**Figure 5:**
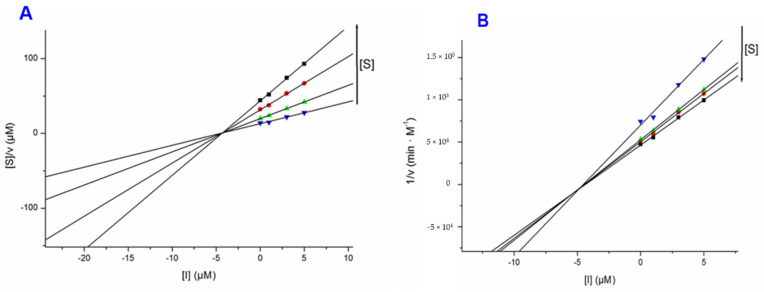
Cornish–Bowden (**A**) and Dixon (**B**) plots for the inhibition of AChE by compound **1**.

Compounds **1** and **5** were screened for their inhibitory effects towards AChE from *Electrophorus electricus* and BChE from equine serum via the Ellman’s method (Table 3 and Figure 5). Galantamine hydrobromide (GH), a clinically used cholinesterase inhibitor was employed as reference compound. The results from the Ellman’s assays showed standard galantamine hydrobromide as a competitive inhibitor for both AChE and BChE (results for the latter not shown). Compound **1** was a strong inhibitor of AChE with a 97.4% inhibition (*K*_i_ = 5.39 µM; *K*_i′_ = 3.54 µM) (Table 3) at a concentration of 10 µM, and acting as a mixed-mode type inhibitor. Compound **1** was a moderate inhibitor of BChE, showing 27.3% inhibition at a concentration of 10 µM.

Compounds **2**, **3** and **5** were tested against α-glucosidase from *Saccharomyces cerevisiae*. Compounds **2** and **5** were not active with 13.4 and 36.1 percentage inhibition, respectively, at concentrations of 10 µM. On the other hand, compound **3** showed a higher inhibitory activity (IC_50_ 164.46 ± 83.04) than the reference compound acarbose (IC_50_ 1072.5 ± 453.2 µM) (see Table 3 and Supplementary Materials Figures S1 and S2). Moreover, compound **5** demonstrated moderate cytotoxic effects towards ovarian carcinoma (A2780: IC_50_ 25.4 µM), colorectal adenocarcinoma (HT29: IC_50_ 20.2 µM), breast cancer (MCF7: IC_50_ 23.7 µM), and thyroid carcinoma (SW1736 IC_50_: 26.2 µM) while the same compound was not active towards pharynx carcinoma (FaDu: IC_50_ > 30 µM). On the other hand, compounds **1-3** were not active (IC_50_ > 30 µM) towards FaDu, A2780, HT29, MCF7, and SW1736.

### 3.3. Docking Study

**Figure 6:**
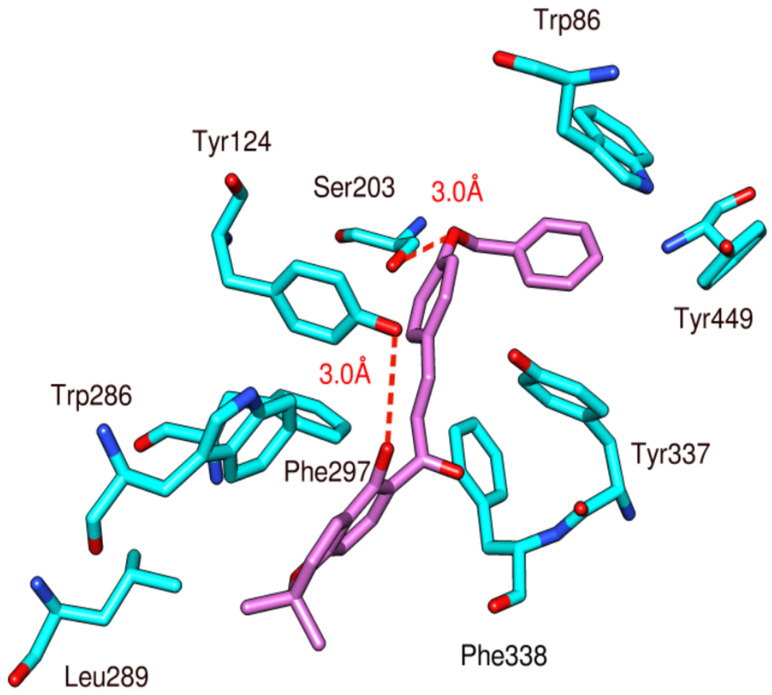
Docking and binding pattern of chalcone **1** (pink) with acetylcholinesterase (cyan).

Docking studies of the natural chalcone **1** against acetylcholinesterase revealed the formation of two hydrogen bonds (Figure 6) that may be responsible for the observed affinity. The oxygen atom of benzyloxy moiety acts as a hydrogen bond acceptor for the side chain of Tyr124 with a bond distance of 3.0 Å. Another hydrogen bond is formed between the OH group of Ser203 and the OH group present at the C-2 position of the phenyl ring with a distance of 3.0 Å. In addition, this compound shows multiple hydrophobic interactions as compared to the standard compound galantamine with various amino acid residues including Trp86, Tyr124, Trp286, Leu289, Phe297, Tyr337, Phe338, and Tyr449.

**Figure 7:**
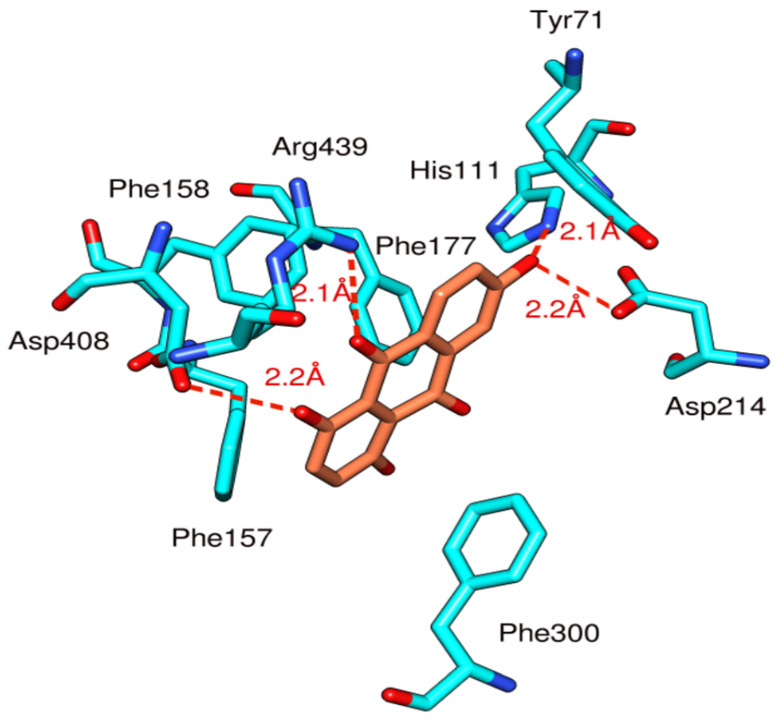
Docking and binding pattern of anthraquinone **3** (orange) with α-glucosidase (cyan).

MOE docking studies of anthraquinone **3** was performed and compared with acarbose, used as a template and the conformation with the lowest energy was chosen for further analysis. Anthraquinone **3** (Figure 7) formed four hydrogen bonds with the His111, Asp408, Arg331, and Arg439. The His111 and the Asp214 were involved in hydrogen bonding with the OH group present at the C-7 position of the anthracene ring with a bond distance of 2.0Å and 2.1 Å, respectively. Similarly, Asp408 formed a hydrogen bond with the OH group attached to C-4 of the anthracene ring of the ligand with a distance of 2.2 Å. Arg439 showed hydrogen bonding with the double bonded oxygen atom present at C-9 of anthracene ring with a bond distance of 2.1 Å. Furthermore, the compound was stabilized in the binding pocket by many hydrophobic interactions with crucial binding site residues including Tyr71, Phe157, Phe158, Phe177, and Phe300, similar to the reference compound acarbose.

## 4. Discussion

*R. muricatus* has been used in various traditional medicinal systems and the current phytochemical investigation lead to isolation of a new chalcone (**1**), a new anthraquinones (**2**), a new-to-nature anthraquinone (**3**), and a new naphthalene analog (**4**), in addition to the previously reported compounds **5–7**. Among these compounds, chalcone **1** showed strong AChE inhibitory activity with 97.4% inhibition at a concentration of 10 µM (*K*i 5.39 ± 0.51 µM and *K*i′ 3.54 ± 0.24 µM), whereas chalcone **5** showed weaker inhibitory activity (49.9 %) at the same concentration. This showed that the benzyl group at C-4 plays a crucial role in the AChE inhibition. Various other natural and synthetic chalcones have been reported as AChE inhibitors [44,45,46,47,48], and chalcones are known to have multiple functions in the treatment of AD, because they inhibit the amyloid beta (Aβ) self-assembly and promotes disassembly of Aβ oligomers [49]. It has been estimated that the number of AD patients will reach 70 million by 2050, due to a rapidly increasing aging population and the unavailability of drugs to cure this disease [50,51,52]. The association between AD and cholinergic neurotransmission deficiency furnishes an excellent base to develop AChE inhibitors as therapeutic drugs [53]. Moreover, the use of AChE inhibitors can relieve some behavioral and cognitive symptoms of AD [54,55].

Compounds **2**, **3**, and **5** were tested for inhibitory activity of α-glucosidase, and it was found that anthraquinone **3** possesses a stronger inhibitory activity than the reference compound acarbose, while compounds **2** and **5** showed no inhibitory activity. Type 2 diabetes is one of the major death causing diseases in the world at present [56]. The incidences of diabetes are rising very rapidly all over the world. Moreover, International Diabetes Federation (IDF) report illustrates that by 2030, the number of diabetic patients will attain 552 million, presenting a global health burden [57]. It is also estimated that diabetes amounts to almost 3.8 million deaths annually, which indicates the poorer glycemic control with current therapies being used for the management of diabetes [58,59]. Different enzymes are involved for the attainment of a normal glucose index in the body. The α-glucosidase enzyme is under extensive study owing to its vitally important role in the management of diabetes mellitus. It is found in the brush border of the small intestine where it hydrolyses and degrades complex carbohydrates into simple monomers and as a result the concentration of glucose is increased in the body [60]. Inhibitors of α-glucosidase thus have the ability to slow down the carbohydrate digestion and absorption in the body. A sudden rise in the glucose level is thus avoided if no insulin is available for the patient [61]. The α-Glucosidase inhibitors have, thus, attracted the interest of researchers for their ability to control diabetes mellitus type 2, and particularly, to manage postprandial hyperglycemia [62].

## 5. Conclusions

Three new compounds (**1**, **2** and **4**), one new-to-nature compound (**3**), and three known compounds **5**–**7** were isolated from *R. muricatus* in this study and tested for their acetylcholinesterase, α-glucosidase and/or cytotoxic effects. Chalcone **1** possesses potent AChE inhibitory activity while anthraquinone **3** showed α-glucosidase effects. A detailed binding mode analysis of the known inhibitors indicated that they may be stabilized in the active site of α-glucosidase and AChE through the simultaneous establishment of multiple hydrogen bonds and hydrophobic interactions. The results demonstrated that the isolated compounds interact efficiently with the active site residues of the respective target enzymes and were found to be similar to that of the reference ligands. The current study thus provides two potentially potent lead compounds, i.e., chalcone **1** and anthraquinone **3,** which can be further developed for the design of novel and efficient drugs for the treatment of Alzheimer’s disease and type 2 diabetes, respectively.

## Figures and Tables

**Table 1:** ^1^H NMR (400 MHz) and ^13^C NMR (100 MHz) data of 4-benzyloxylonchocarpin (**1**) and muracatane A (**2**).

	1			2	
No	δ_H_ (nH, Multiplicity, *J* in Hz)	δ_C_	No	δ_H_ (nH, Multiplicity, *J* in Hz)	δ_C_
1′	-	109.4	1	-	158.7
2′	-	160.9	2	-	138.4
3′	-	114.1	3	7.60 (1H, d, *J* = 8.0 Hz)	136.4
4′	-	159.6	4	8.00 (1H, d, *J* = 8.0 Hz)	123.2
5′	6.37 (1H, d, *J* = 9.0 Hz)	108.1	4a	-	137.0
6′	7.72 (1H, d, *J* = 9.0 Hz)	130.5	5	-	159.8
α	7.45 *(*1H, d, *J* = 16 Hz, 1H)	127.8	6	7.30 (1H, dd, *J* = 8.0, 2.0 Hz)	117.1
β	7.86 (1H, d, *J* = 16.0 Hz, 1H)	144.0	7	7.70 (1H, t, *J* = 8.0 Hz)	135.0
keto	-	191.9	8	7.90 (1H, dd, *J* = 8.0, 2.0 Hz)	119.6
OH-2′	13.77		8a	-	137.0
1	-	128.0	9	-	182.7
2,6	7.60 (2H, BB′)	130.3	9a	-	124.8
3,5	7.00 (2H, AA′)	115.3	10	-	182.1
4	-	160.9	10a	-	120.9
1′′	6.76 (1H, d, *J* = 10 Hz)	115.9	1′	3.34 (1H, d, *J* = 8.0 Hz)	30.1
2′′	5.59 (1H, d, *J* = 10 Hz)	127.4	2′	3.90 (1H, t, *J* = 8.0 Hz)	51.3
3′′	-	77.7	CO_2_Me	3.70 (6H, s)	52.6
4′′,5′′	1.47 (6H, s)	28.3	CO_2_Me	-	169.0
1′′′	-	136.3	1-OMe	3.97 (3H, s)	62.0
2′′′–6′′′	7.46 (2H, m)	127.4	5-OMe	4.04 (3H, s)	56.3
3′′′,5′′′	7.40 (2H, m)	128.6			
4′′′	7.32 (1H, m)	127.8			
*CH_2_*Bn	5.12 (2H, s)	70.1			

**Table 2:** ^1^H NMR (400 MHz) and ^13^C NMR (100 MHz) data of muracatane C (**3**) and D (**4**).

	3			4	
No	δ_H_ (nH, Multiplicity, *J* in Hz)	δ_C_	No	δ_H_ (nH, Multiplicity, *J* in Hz)	δ_C_
1	-	156.4	1	-	117.9
2	7.28 (1H, m)	128.3	2	-	154.2
3	7.28 (1H, m)	129.2	3	7.09 (1H, d, *J* = 8.0 Hz)	111.0
4		156.5	4	7.56 (1H, d, *J* = 8.0 Hz)	125.9
4a		112.0	4a		124.3
5	7.98 (1H, d, *J* = 8.0 Hz)	129.5	5	7.49 (1H, s)	129.1
6	7.20 (1H, dd, *J* = 8.0, 2.0 Hz)	121.8	6	-	125.6
7		163.6	7	-	157.2
8	7.40 (1H, d, *J* = 2.0 Hz)	112.1	8	7.08 (1H, s)	100.3
8a	-	134.8	8a	-	133.5
9	-	186.3	1-Me	2.50 (3H, s)	10.7
9a	-	112.5	6-Me	2.33 (3H, s)	16.5
10	-	185.3	7-OMe	3.95 (3H, s)	55.1
10a	-	124.3	2-OMe	3.91 (3H, s)	56.6
OH-1	12.53 (1H, s)	-			
OH-4	12.84 (1H, s)	-			
OH-7	11.18 (1H, s)	-			

**Table 3:** Inhibitory activities of isolated compounds **1**–**5** towards cholinesterases and α-glucosidase.

	AChE		BChE	α-Glucosidase	
Compound	% Inhibition ^a^	*K*_i_ (µM) (*K*_i_’ (µM))	% Inhibition ^a^	% Inhibition ^a^	IC_50_ (µM)
1	97.4	5.39 ± 0.51 (3.54 ± 0.24)	27.3	n.d.	n.d.
2	n.d.	n.d.	n.d.	13.4	n.d.
3	n.d.	n.d.	n.d.	80.4	164.46 ± 83.04
4	n.d.	n.d.	n.d.	n.d.	n.d.
5	49.9	n.d.	8.7	36.1	n.d.
GH	90.5	0.54	54.8	Acarbose	1072.5 ± 453.2

^a^ % Inhibition at concentration of 10 μM. AChE, acetylcholinesterase; BChE, butyrylcholinesterase. n.d. = not determined.

## Supplementary Materials

The following are available online at https://www.mdpi.com/2218-273X/10/11/1562/s1: copies of 1D (^1^H, ^13^C), 2D NMR, and mass spectra of compounds **1**–**4**.
