# Supplementary material for: 4-Benzyloxylonchocarpin and Muracatanes A-C from Ranunculus muricatus L. and Their Biological Effects
Source: Biomolecules. 2020 Nov 17;10(11):1562. doi: 10.3390/biom10111562 (PMC7698453; doi:10.3390/biom10111562)
Supplement: Supplementary file 1 [file biomolecules-10-01562-s001.pdf]

Supplementary Information

4-Benzyloxylonchocarpin and Muracatanes A-C from *Ranunculus muricatus* L. and Their Biological Effects

Hidayat Hussain [1,*], Iftikhar Ali [2,3], Daijie Wang [2], Nilufar Z. Mamadalieva [1], Wahid Hussain [4], René Csuk [5], Anne Loesche [5], Lucie Fischer [5], Dan Staerk [6], Syariful Anam [6,7], Mashail N. AlZain [8, *], Maria Mushtaq [9], Zaheer Ul-Haq [9], Riaz Ullah [10], Omar M. Noman [10], Ghulam Abbas [11] and Ivan R. Green [12]

1 Department of Bioorganic Chemistry, Leibniz Institute of Plant Biochemistry, Weinberg 3, D-06120 Halle (Salle), Germany; Nilufar.Mamadalieva@ipb-halle.de (N.Z.M.)
2 School of Pharmaceutical Sciences and Key Laboratory for Applied Technology of Sophisticated Analytical Instruments of Shandong Province, Shandong Analysis and Test Center, Qilu University of Technology (Shandong Academy of Sciences), Jinan 250014, China; iftikhar.ali@kiu.edu.pk (I.A.); wangdaijie@qlu.edu.cn (D.W.)
3 Department of Chemistry, Karakoram International University, Gilgit 15100, Pakistan
4 Department of Botany, Government Post Graduate College Parachinar, Parachinar 26300, District Kurram, Pakistan; wahidhussain@uop.edu.pk
5 Martin-Luther University Halle-Wittenberg, Organic Chemistry, Kurt-Mothes-Str. 2, D-06120, Halle, Saale, Germany; rene.csuk@chemie.uni-halle.de (R.C.); anne.loesche@web.de (A.L.); lucie.fischer2018@gmx.de (L.F.)
6 Department of Drug Design and Pharmacology, Faculty of Health and Medical Sciences, University of Copenhagen, Copenhagen, Denmark; ds@sund.ku.dk (D.S.); syarifulanam1@gmail.com (S.A.)
7 Department of Pharmacy, Faculty of Sciences, Tadulako University, Palu 94118, Indonesia
8 Department of Biology, College of Sciences, Princess Nourah Bint Abdulrahman University, Riyadh 11451, Saudi Arabia
9 Dr. Panjwani Center for Molecular Medicine and Drug Research, International Center for Chemical and Biological Sciences, University of Karachi, Karachi-75270, Pakistan; mariahasnain@gmail.com (M.M.) zaheer.qasmi@iccs.edu (Z.U.)
10 Department of Pharmacognosy (MAPPRC), College of Pharmacy, King Saud University, P.O. Box 2457, Riyadh 11451, Saudi Arabia; rullah@ksu.edu.sa (R.U.); onoman@ksu.edu.sa (O.M.N.)
11 Department of Biological Sciences and Chemistry, College of Arts and Sciences, University of Nizwa, Nizwa-616, Sultanate of Oman; abbashej@unizwa.edu.om
12 Department of Chemistry and Polymer Science, University of Stellenbosch, Private Bag X1, Matieland, Stellenbosch 7600, South Africa; irg@sun.ac.za
* Correspondence: hussainchem3@gmail.com/Hidayat.Hussain@ipb-halle.de (H.H.); mnalzain@pnu.edu.sa (M.N.A.)

Contents

Page S3: Figure S1. The α-glucosidase IC_{50} curve of muracatane B (**3**)
Page S4: Figure S2. The α-glucosidase IC_{50} curve of acarbose
Page S5: Figure S3. ^{1}H NMR spectrum of 4-benzyloxylonchocarpin (**1**) (400 MHz, $CDCl_3$).

Page S6: Figure S4. ^{13}C NMR spectrum of 4-benzyloxylonchocarpin (**1**) (400 MHz, $CDCl_3$).

Page S7: Figure S5. COSY spectrum of 4-benzyloxylonchocarpin (**1**) (400 MHz, $CDCl_3$).

Page S8: Figure S6. HSQC spectrum of 4-benzyloxylonchocarpin (**1**) (400 MHz, $CDCl_3$).

Page S9: Figure S7. HMBC spectrum of 4-benzyloxylonchocarpin (**1**) (400 MHz, $CDCl_3$).

Page S10: Figure S8. HRESIMS spectrum of 4-benzyloxylonchocarpin (**1**).

Page S11: Figure S9. ^{1}H NMR spectrum of muracatane A (**2**) (400 MHz, $CDCl_3$).

Page S12: Figure S10. ^{13}C NMR spectrum of muracatane A (**2**) (400 MHz, $CDCl_3$).

Page S13: Figure S11. DEPT spectrum of muracatane A (**2**) (400 MHz, $CDCl_3$).

Page S14: Figure S12. COSY spectrum of muracatane A (**2**) (400 MHz, $CDCl_3$).

Page S15: Figure S13. HSQC spectrum of muracatane A (**2**) (400 MHz, $CDCl_3$).

Page S16: Figure S14. HMBC spectrum of muracatane A (**2**) (400 MHz, $CDCl_3$).

Page S17: Figure S15. HRESIMS spectrum of muracatane A (**2**).

Page S18: Figure S16. ^{1}H NMR spectrum of muracatane B (**3**) (400 MHz, $CDCl_3$).

Page S19: Figure S17. ^{13}C NMR spectrum of muracatane B (**3**) (400 MHz, $CDCl_3$).

Page S20: Figure S18. COSY spectrum of muracatane B (**3**) (400 MHz, $CDCl_3$).

Page S21: Figure S19. HSQC spectrum of muracatane B (**3**) (400 MHz, $CDCl_3$).

Page S22: Figure S20. HMBC spectrum of muracatane B (**3**) (400 MHz, $CDCl_3$).

Page S23: Figure S21. HRESIMS spectrum of muracatane B (**3**).

Page S24: Figure S22. ^{1}H NMR spectrum of muracatane C (**4**) (400 MHz, $CDCl_3$).

Page S25: Figure S23. ^{13}C NMR spectrum of muracatane C (**4**) (400 MHz, $CDCl_3$).

Page S26: Figure S24. DEPT spectrum of muracatane C (**4**) (400 MHz, $CDCl_3$).

Page S27: Figure S25. COSY spectrum of muracatane C (**4**) (400 MHz, $CDCl_3$).

Page S28: Figure S26. HSQC spectrum of muracatane C (**4**) (400 MHz, $CDCl_3$).

Page S29: Figure S27. HMBC spectrum of muracatane C (**4**) (400 MHz, $CDCl_3$).

Page S30: Figure S28. HRESIMS spectrum of muracatane C (**4**).

Page S31: Figure S29. ^{1}H NMR spectrum of 4-methoxylonchocarpin (**5**)

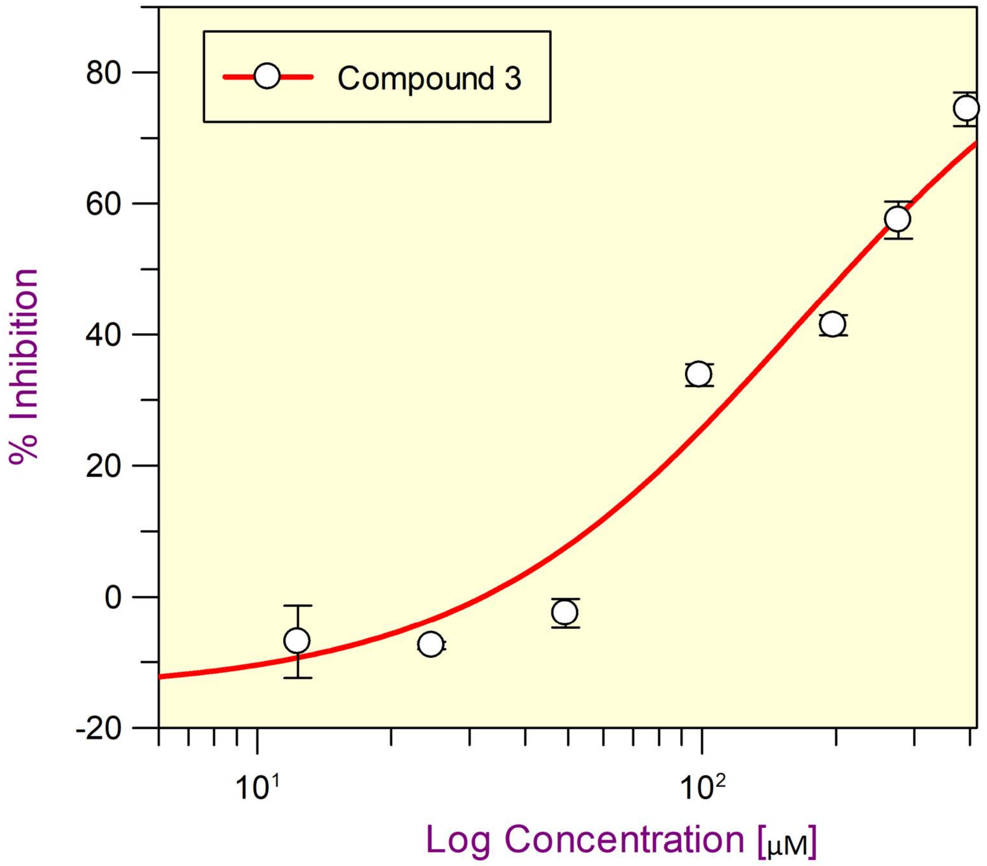

Parameter	Value	Std. Error
Y Range	112.8954	46.5382
IC 50	166.3200	83.9814
Slope factor	-1.1616	1.2098
Background	-14.5747	12.0302

Page S3: Figure S1. The α-glucosidase IC_{50} curve of muracatane B (**3**)

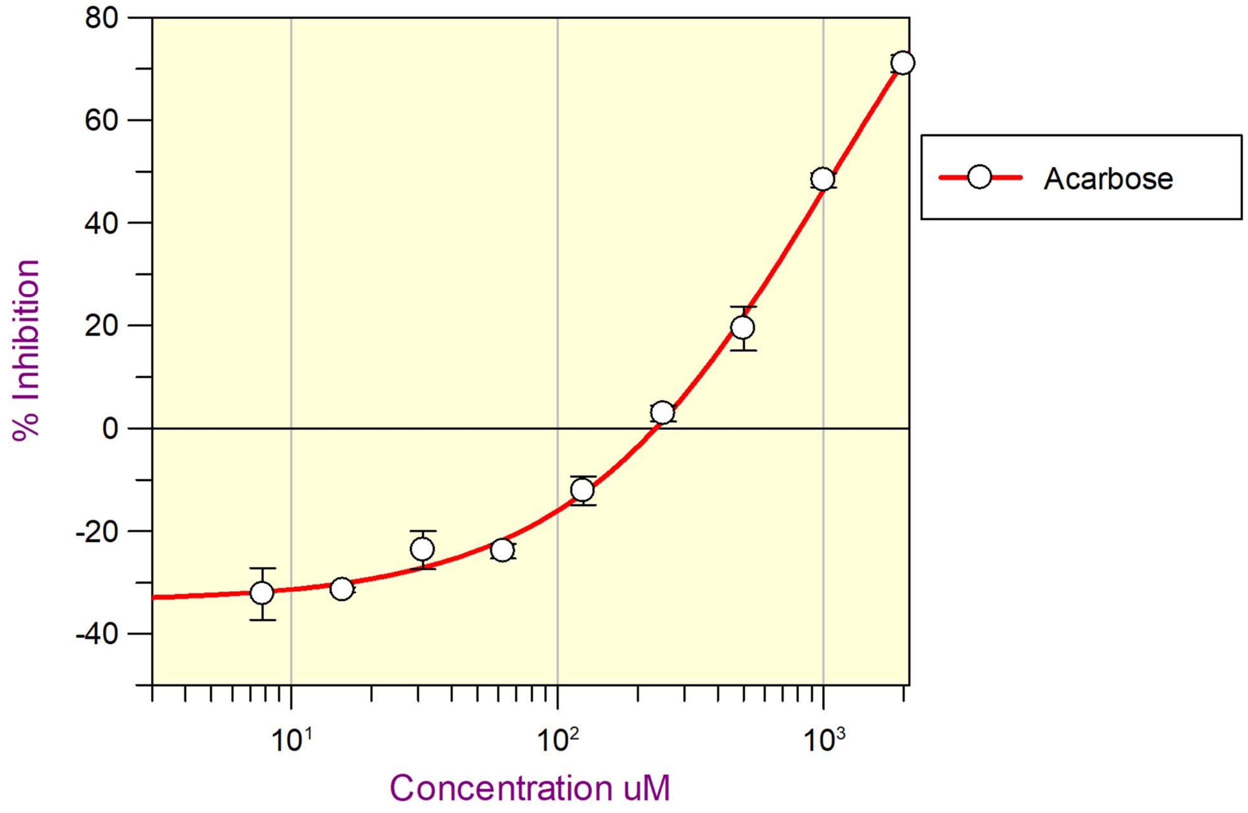

Parameter	Value	Std. Error
Y Range	165.8226	32.8196
IC 50	1072.5090	453.2152
Slope factor	-0.8921	0.1458
Background	-33.8514	2.6533

Page S4: Figure S2. The α-glucosidase IC_{50} curve of acarbose

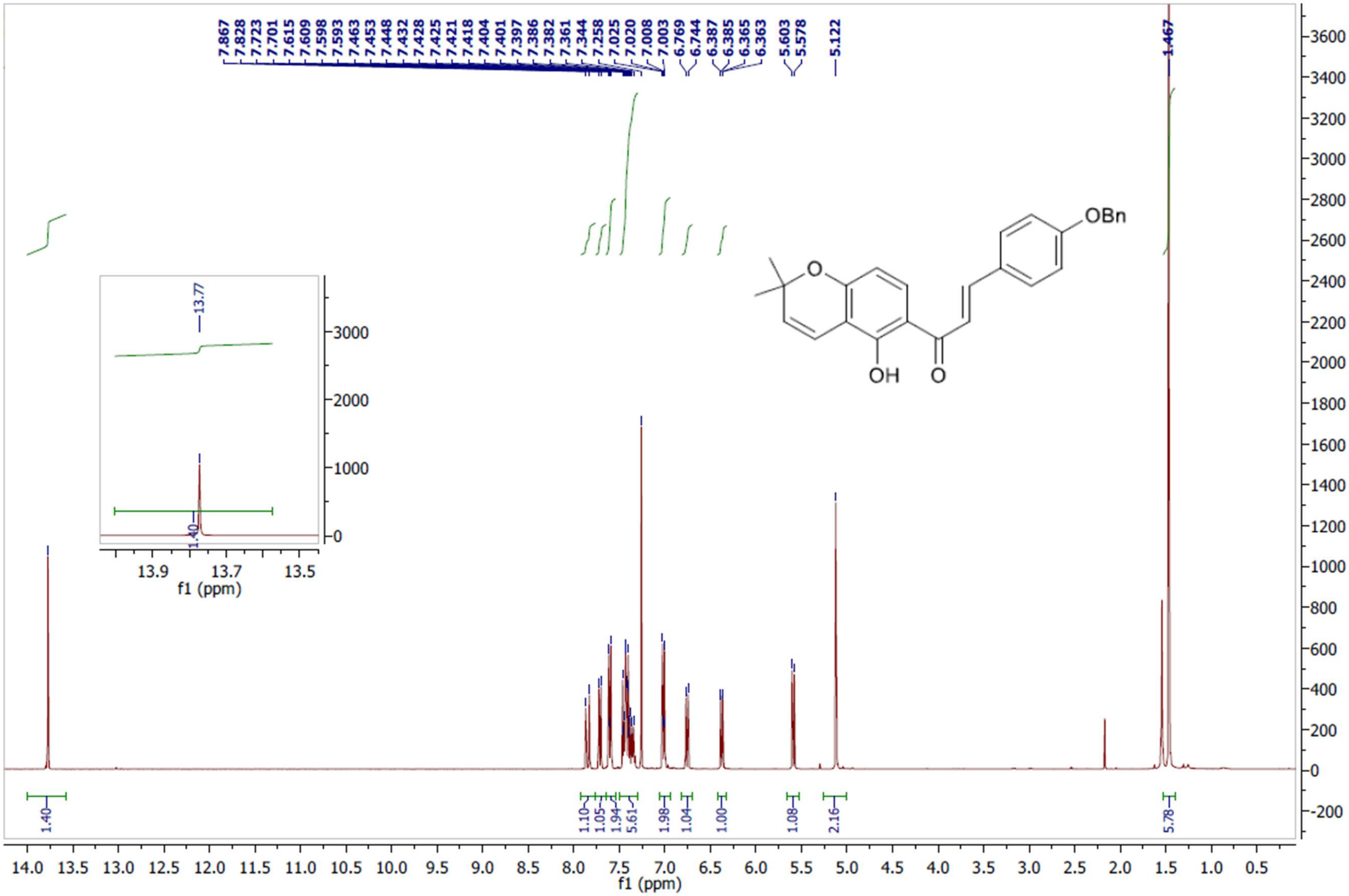

Page S5: Figure S3. ^{1}H NMR spectrum of 4-benzyloxylonchocarpin (**1**) (400 MHz, $CDCl_3$).

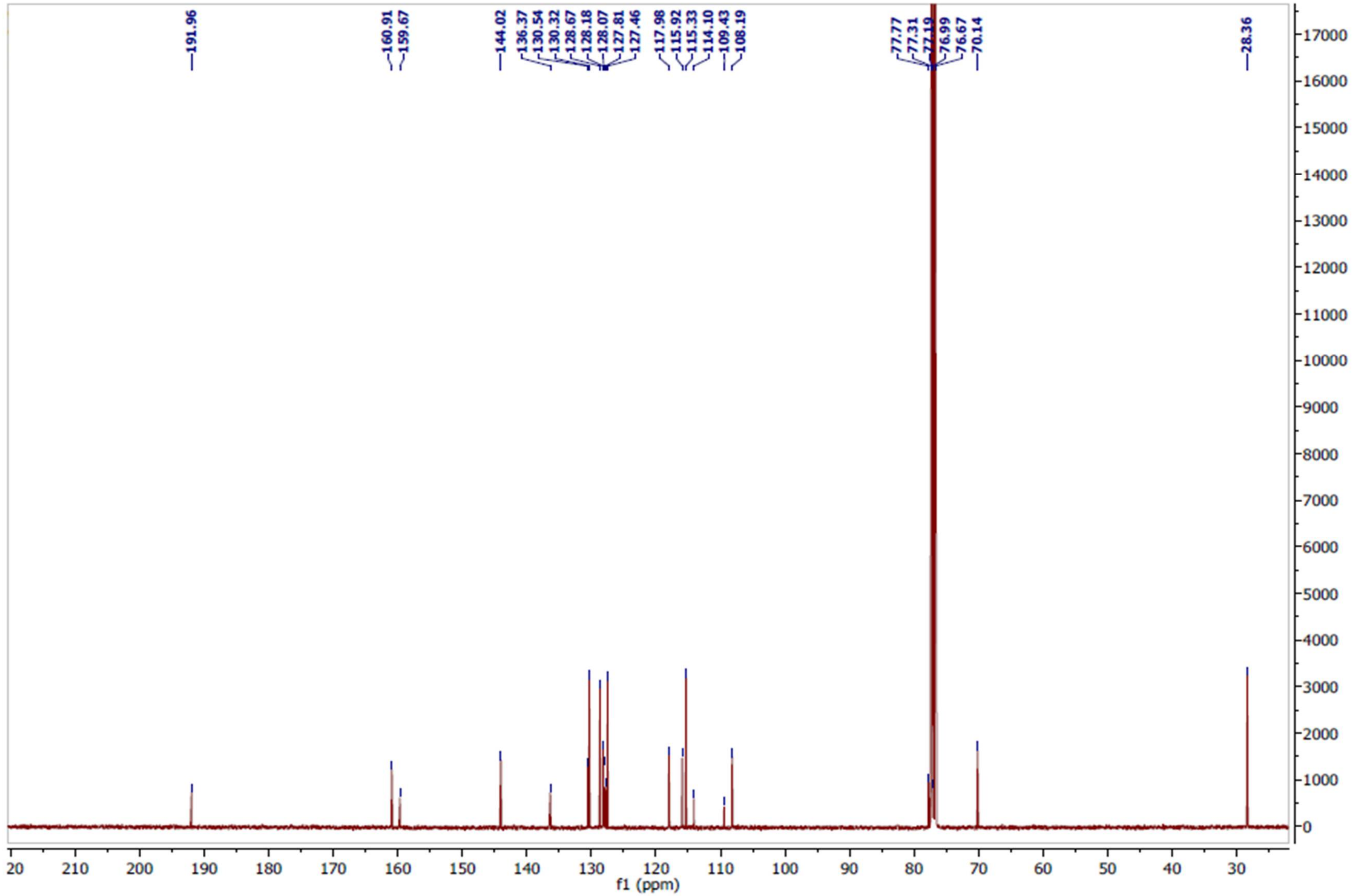

Page S6: Figure S4. ^{13}C NMR spectrum of 4-benzyloxylonchocarpin (**1**) (400 MHz, $CDCl_3$).

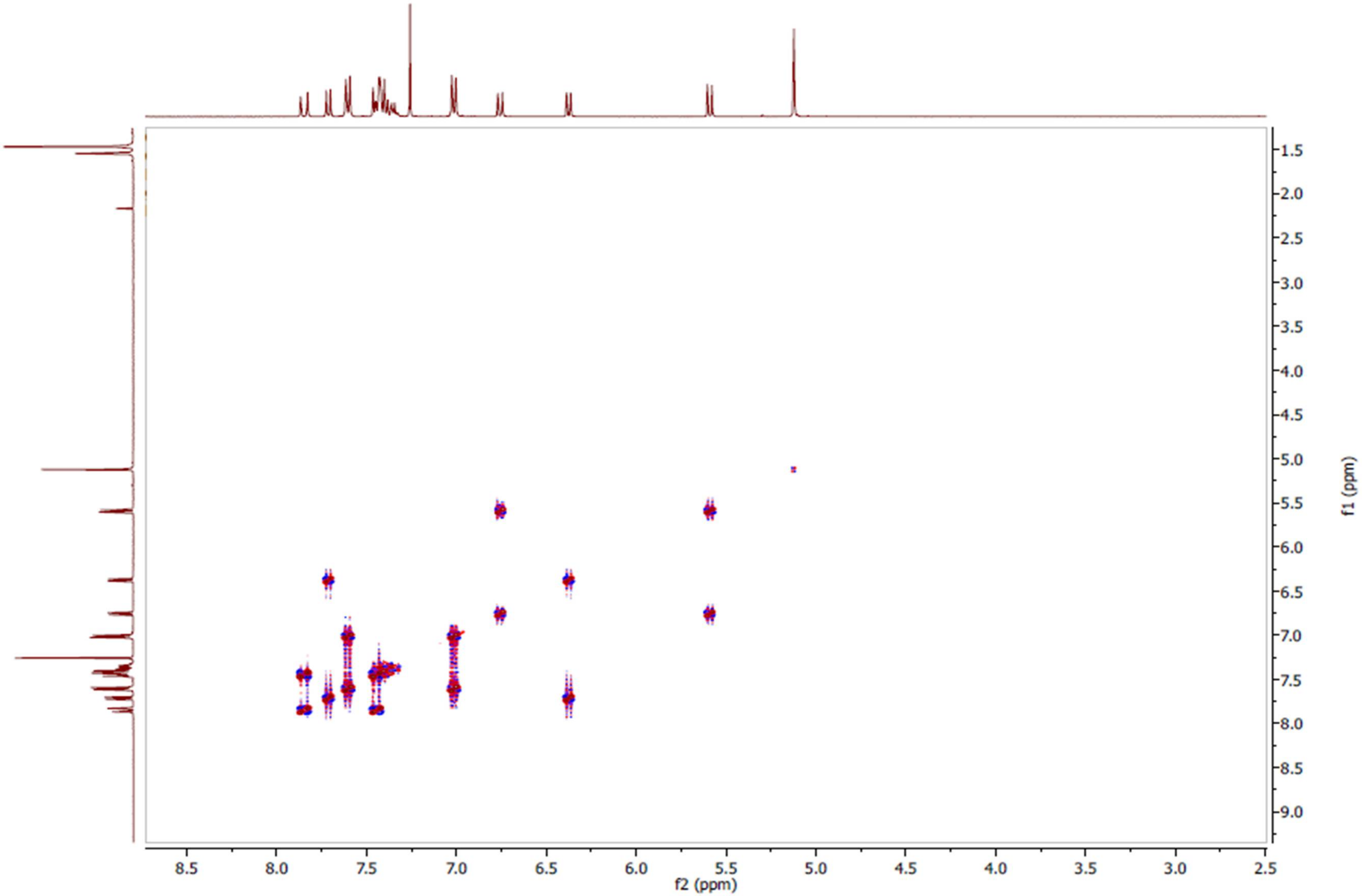

Page S7: Figure S5. COSY spectrum of 4-benzyloxylonchocarpin (**1**) (400 MHz, $CDCl_3$).

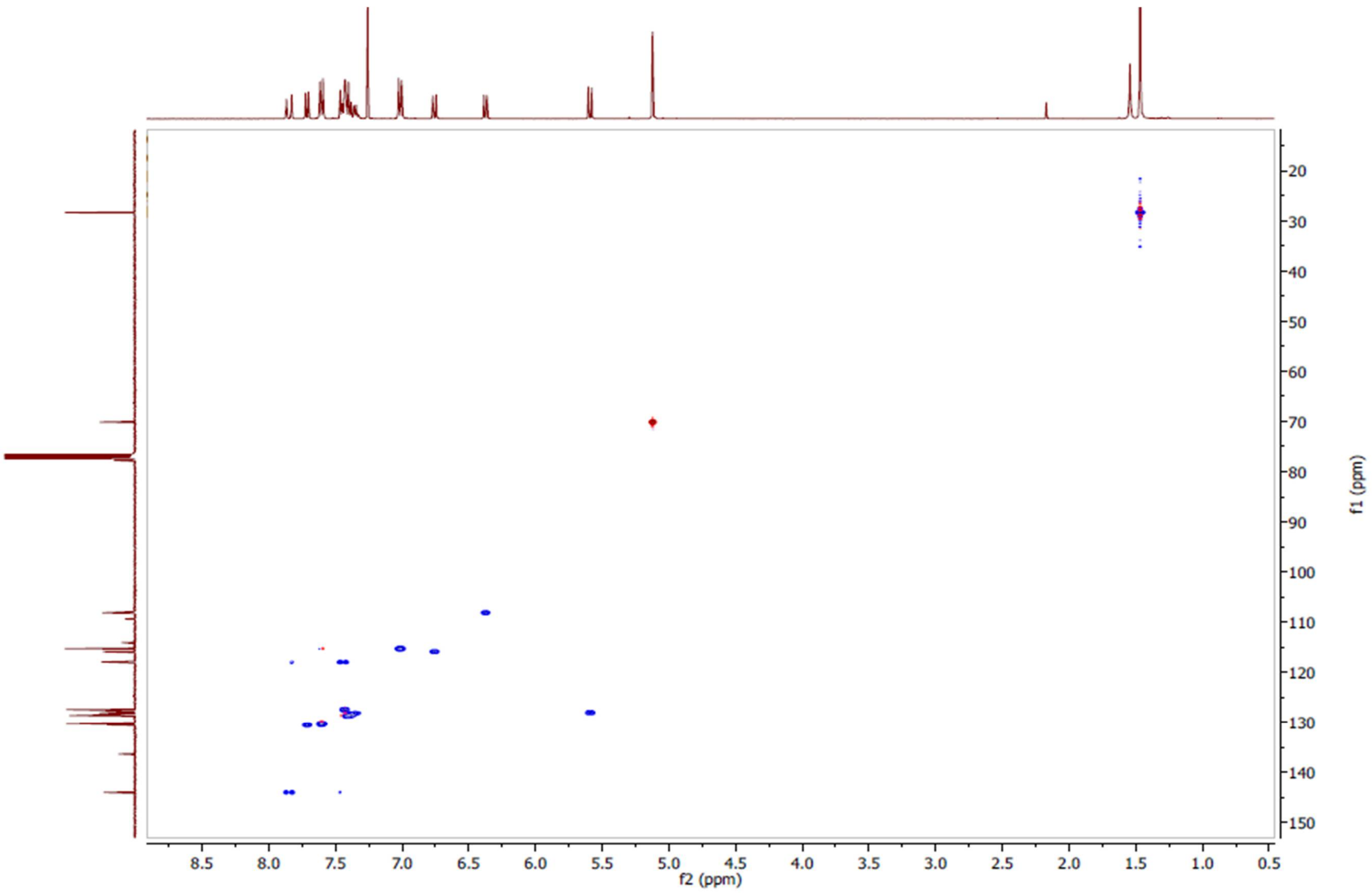

Page S8: Figure S6. HSQC spectrum of 4-benzyloxylonchocarpin (**1**) (400 MHz, $CDCl_3$).

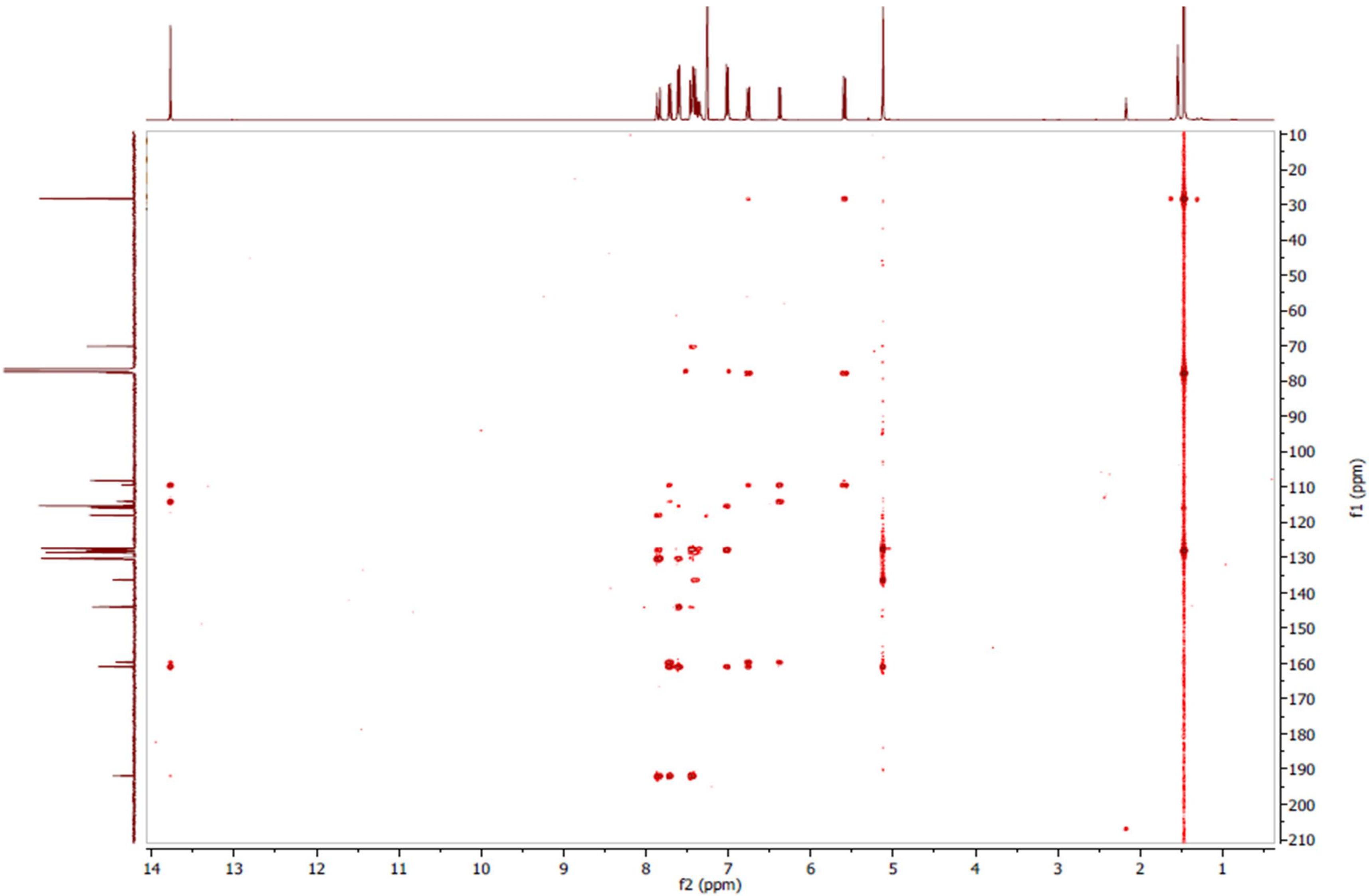

Page S9: Figure S7. HMBC spectrum of 4-benzyloxylonchocarpin (**1**) (400 MHz, $CDCl_3$).

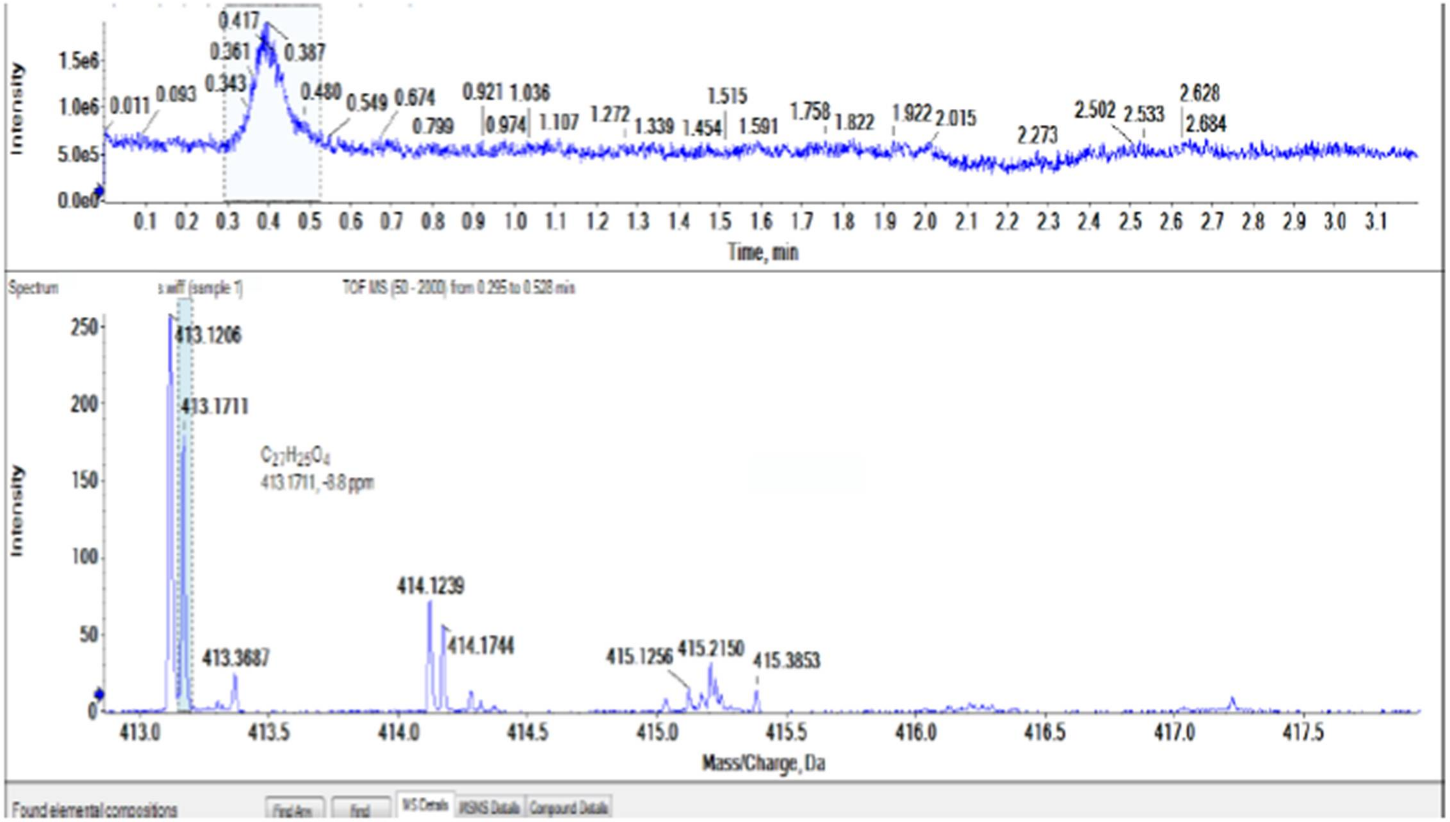

Page S10: Figure S8. HRESIMS spectrum of 4-benzyloxylonchocarpin (**1**).

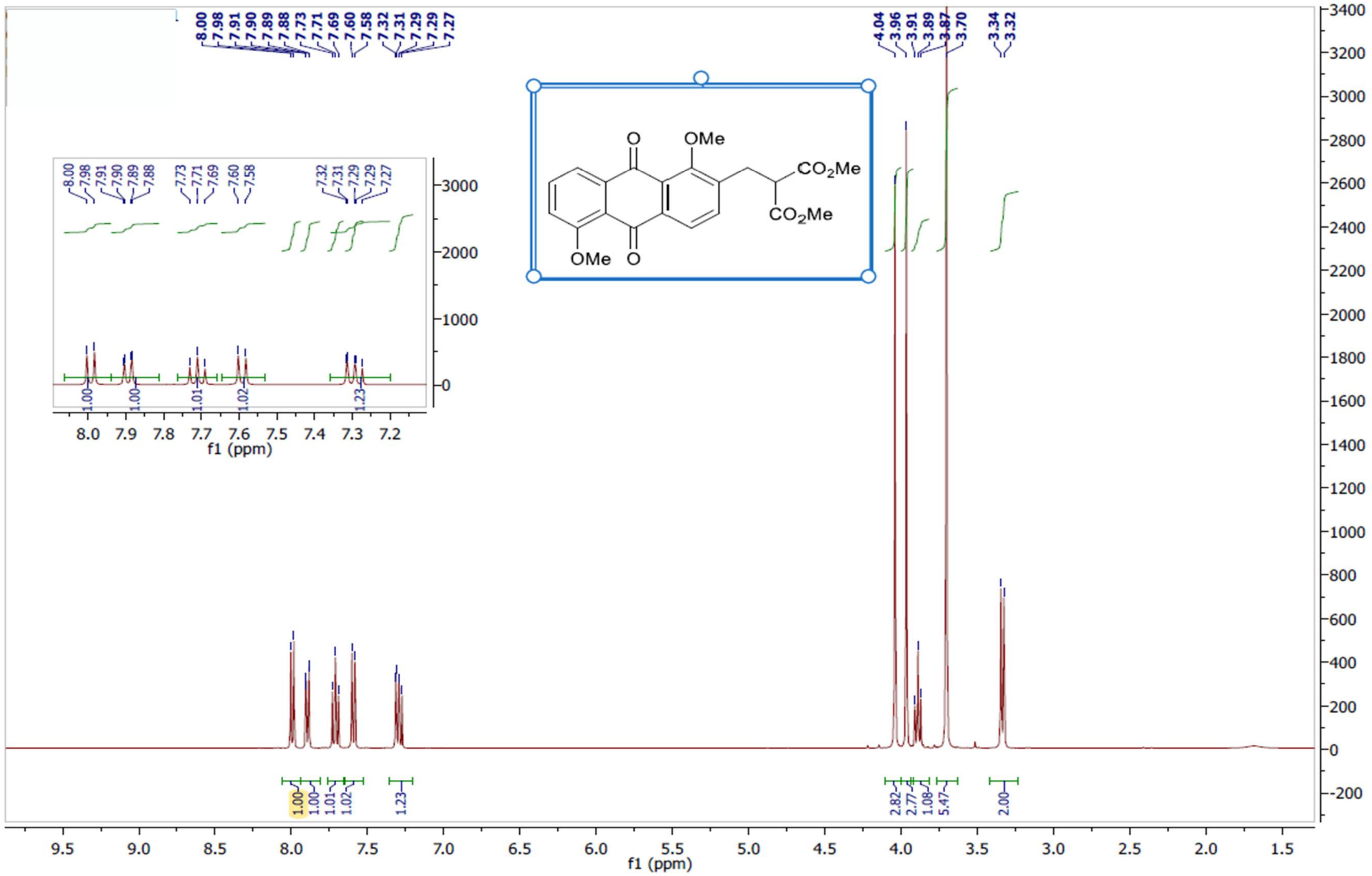

Page S11: Figure S9. ^{1}H NMR spectrum of muracatane A (**2**) (400 MHz, $CDCl_3$).

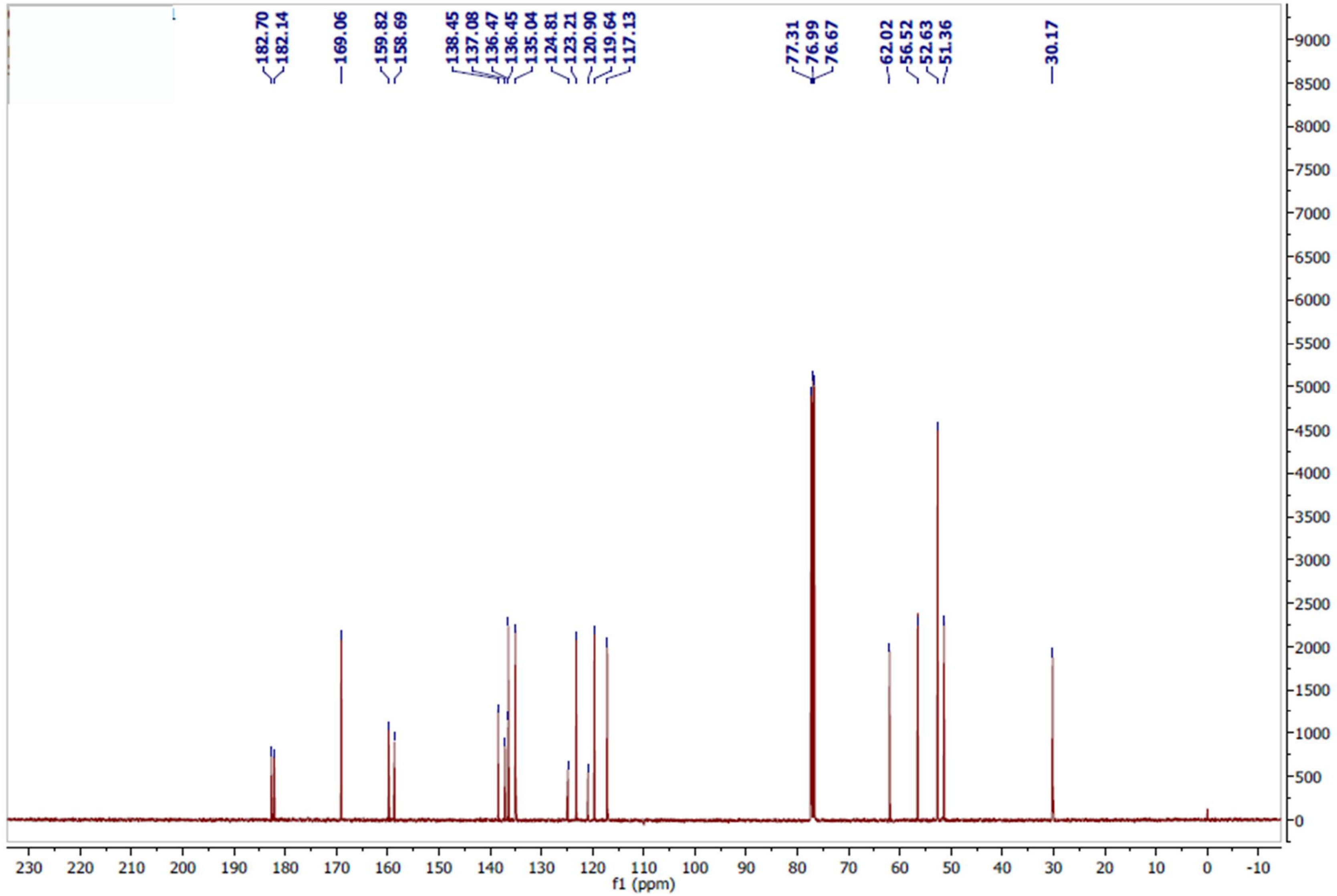

Page S12: Figure S10. ^{13}C NMR spectrum of muracatane A (**2**) (400 MHz, $CDCl_3$).

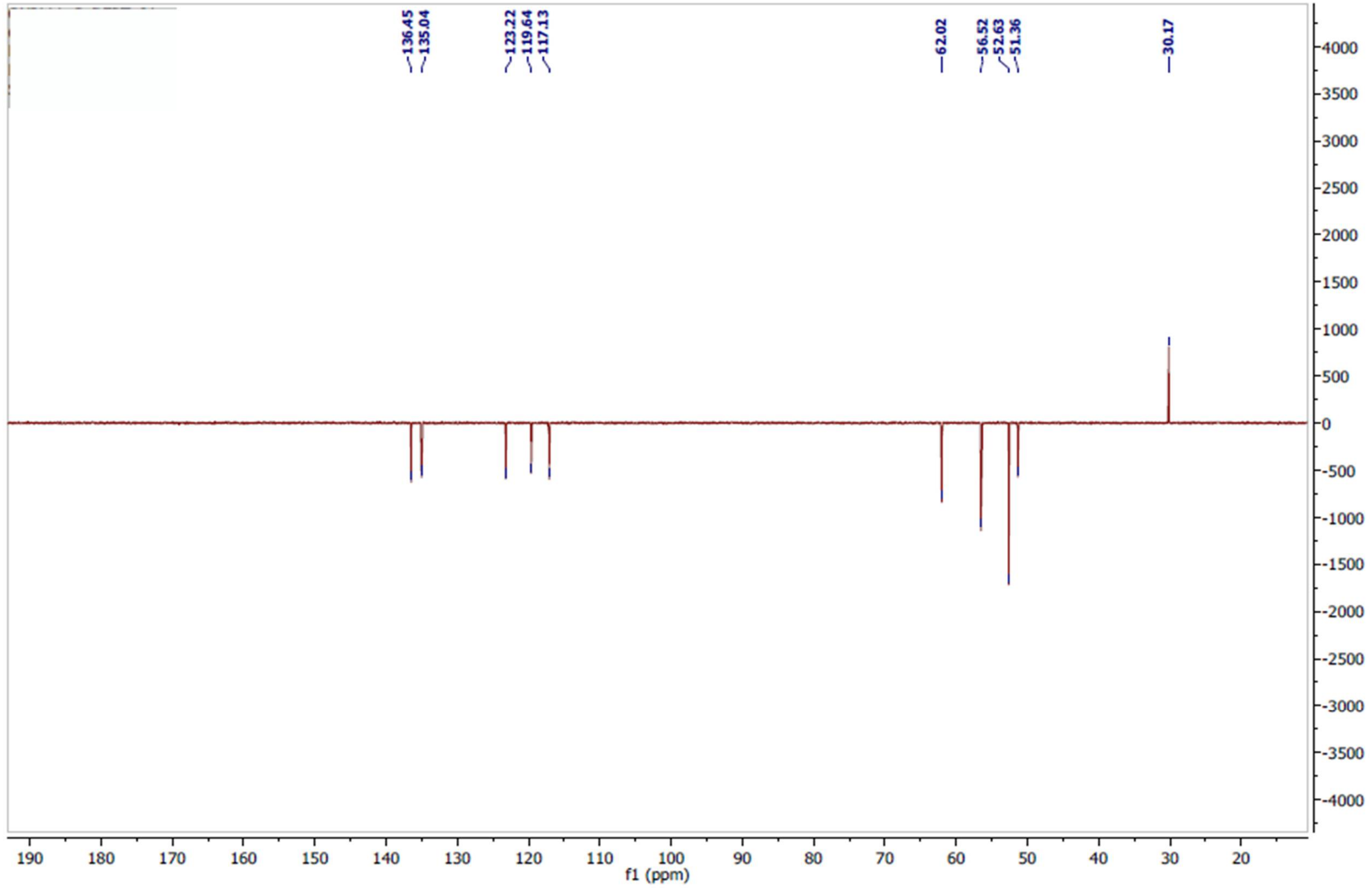

Page S13: Figure S11. DEPT spectrum of muracatane A (**2**) (400 MHz, $CDCl_3$).

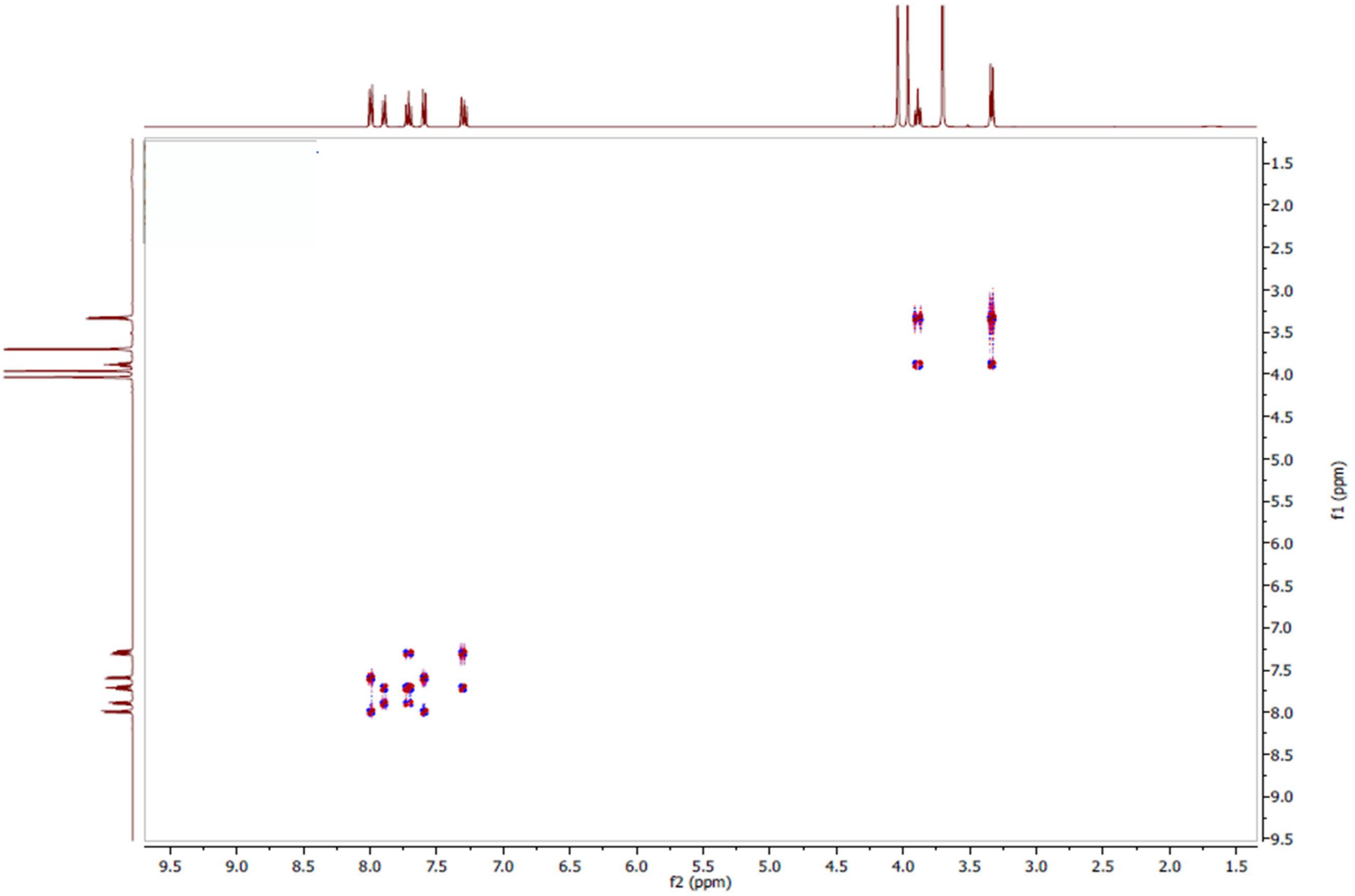

Page S14: Figure S12. COSY spectrum of muracatane A (**2**) (400 MHz, $CDCl_3$).

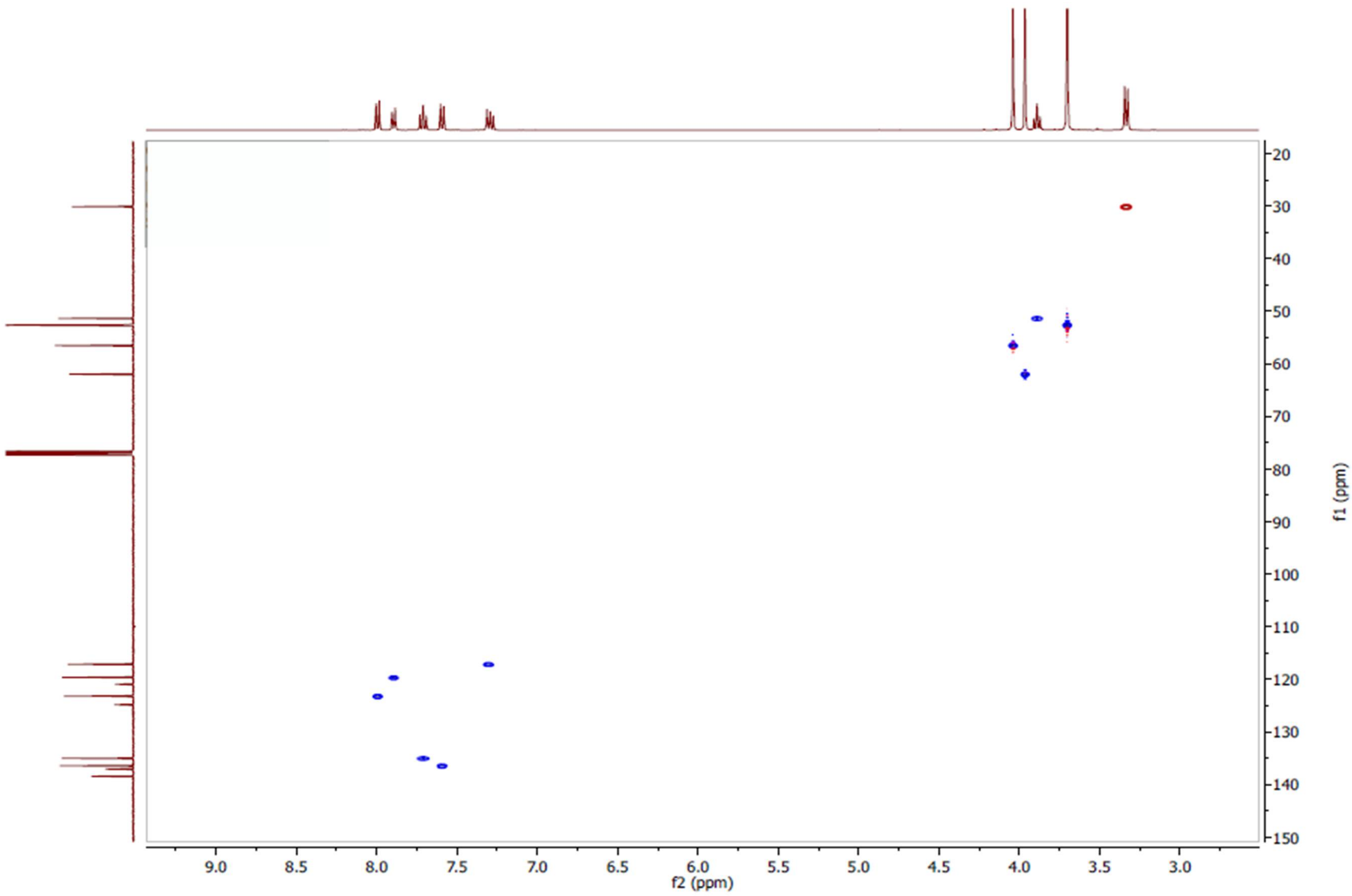

Page S15: Figure S13. HSQC spectrum of muracatane A (**2**) (400 MHz, $CDCl_3$).

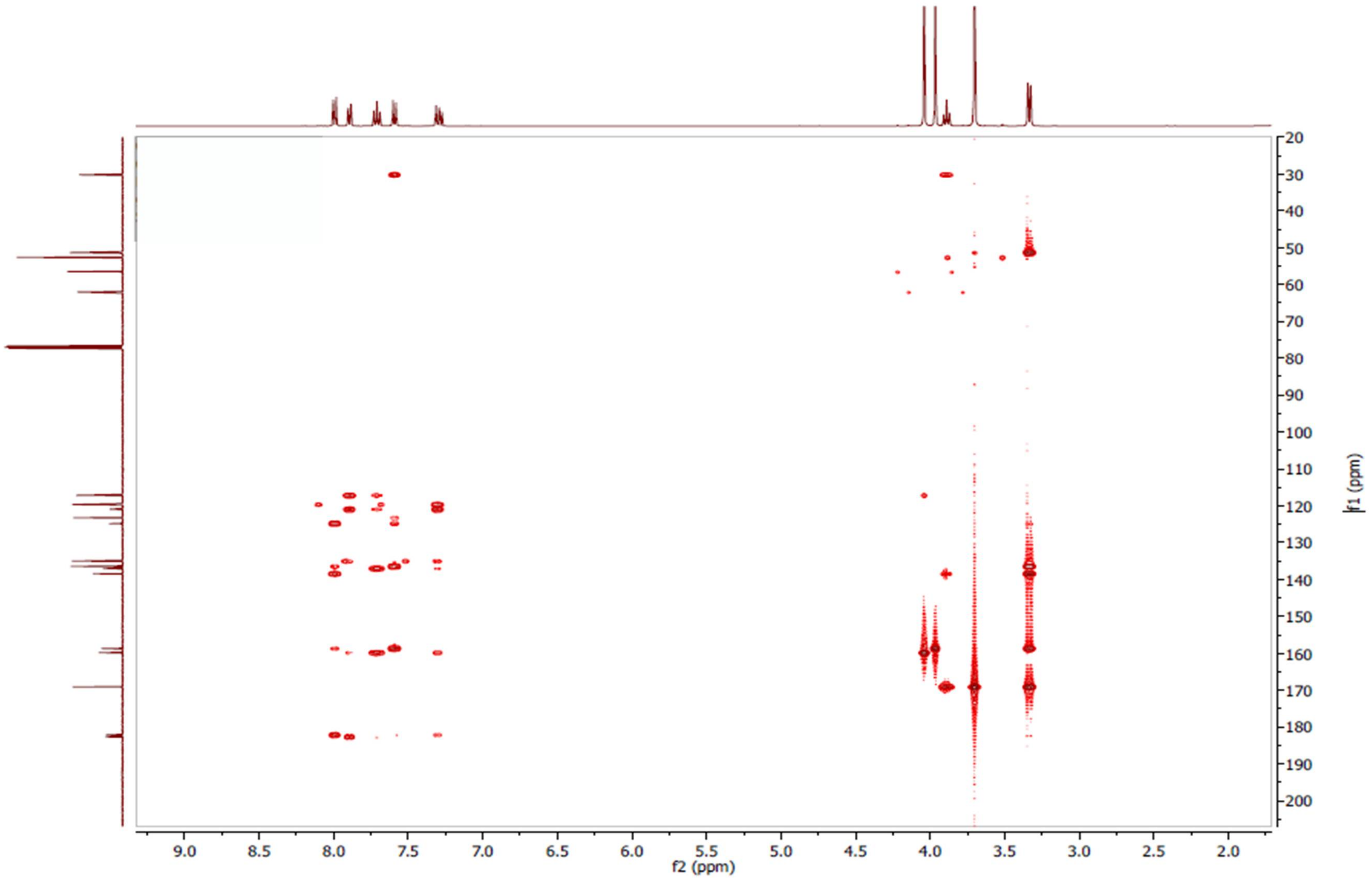

Page S16: Figure S14. HSQC spectrum of muracatane A (**2**) (400 MHz, $CDCl_3$).

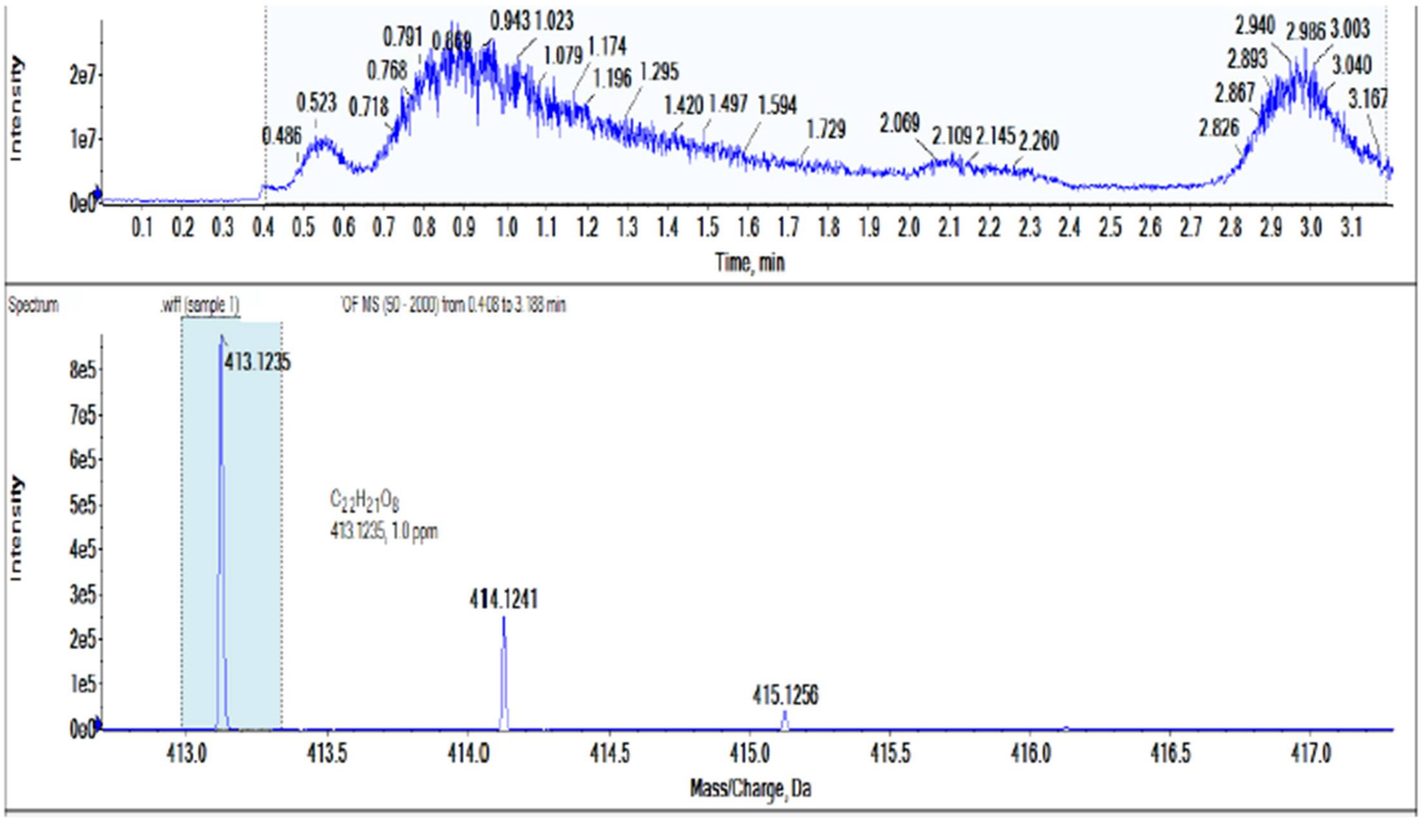

Page S17: Figure S15. HRESIMS spectrum of muracatane A (**2**).

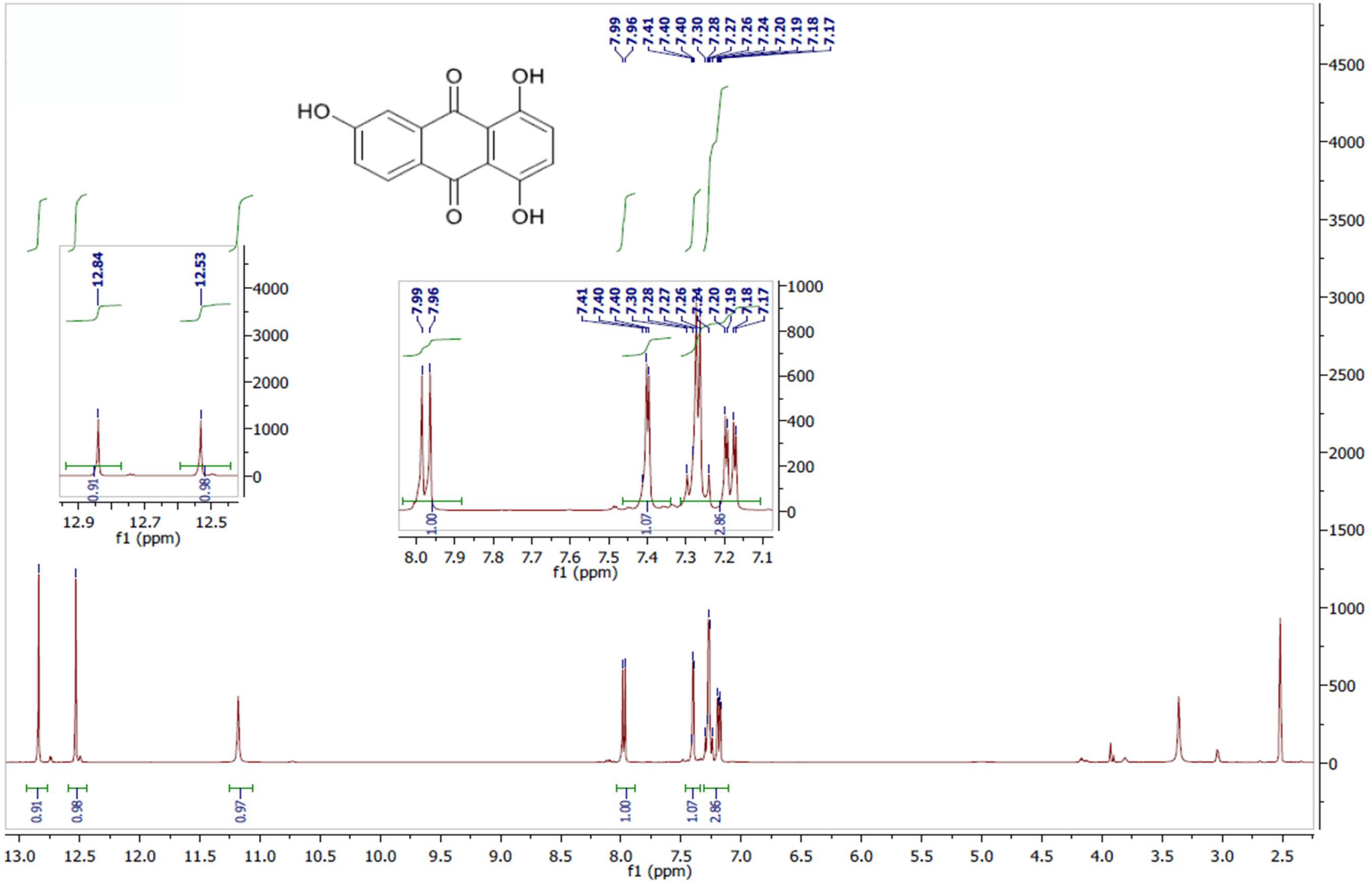

Page S18: Figure S16. ^{1}H NMR spectrum of muracatane B (**3**) (400 MHz, DMSO-d_6).

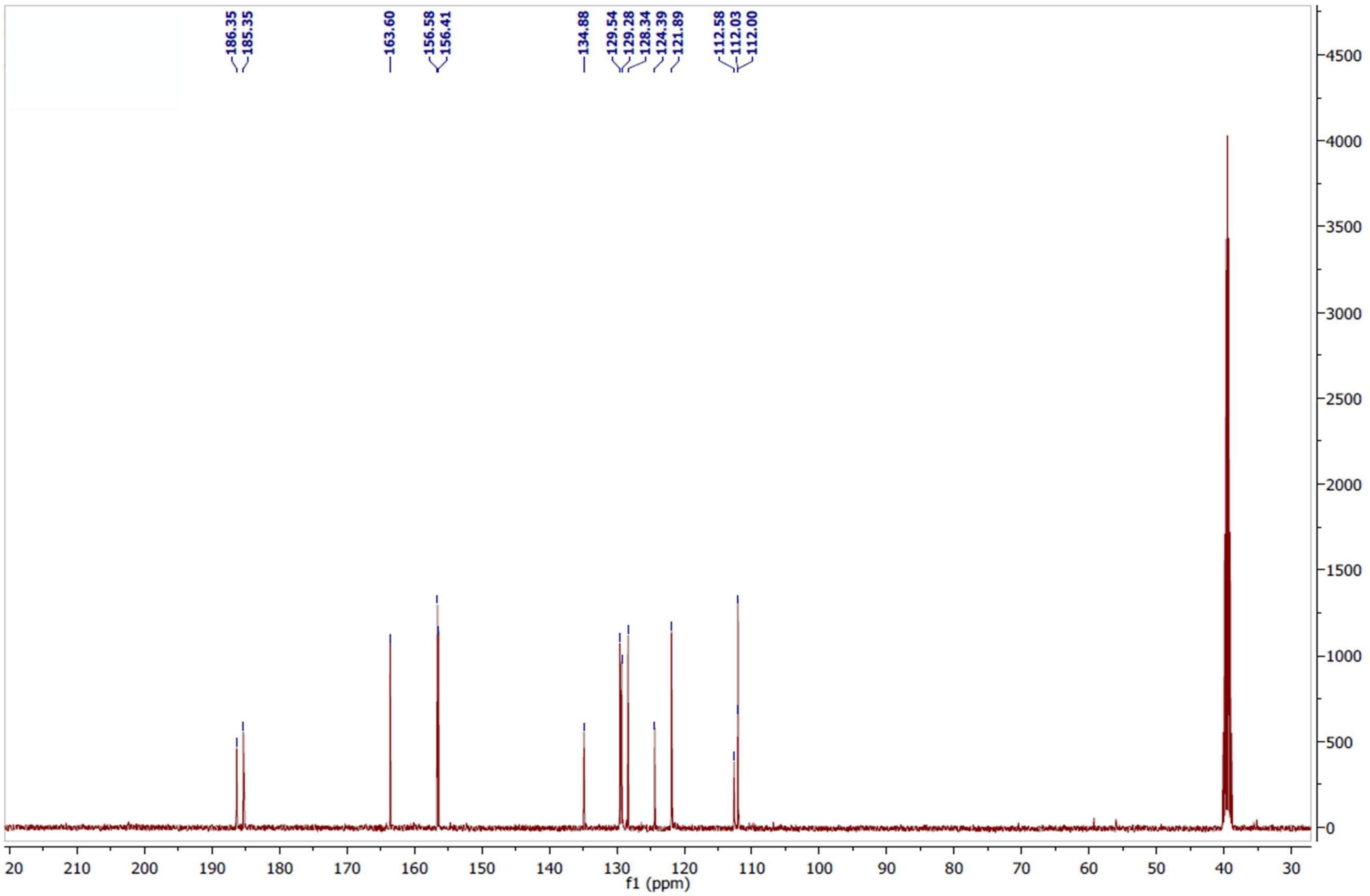

Page S19: Figure S17. ^{13}C NMR spectrum of muracatane B (**3**) (400 MHz, DMSO-d_6).

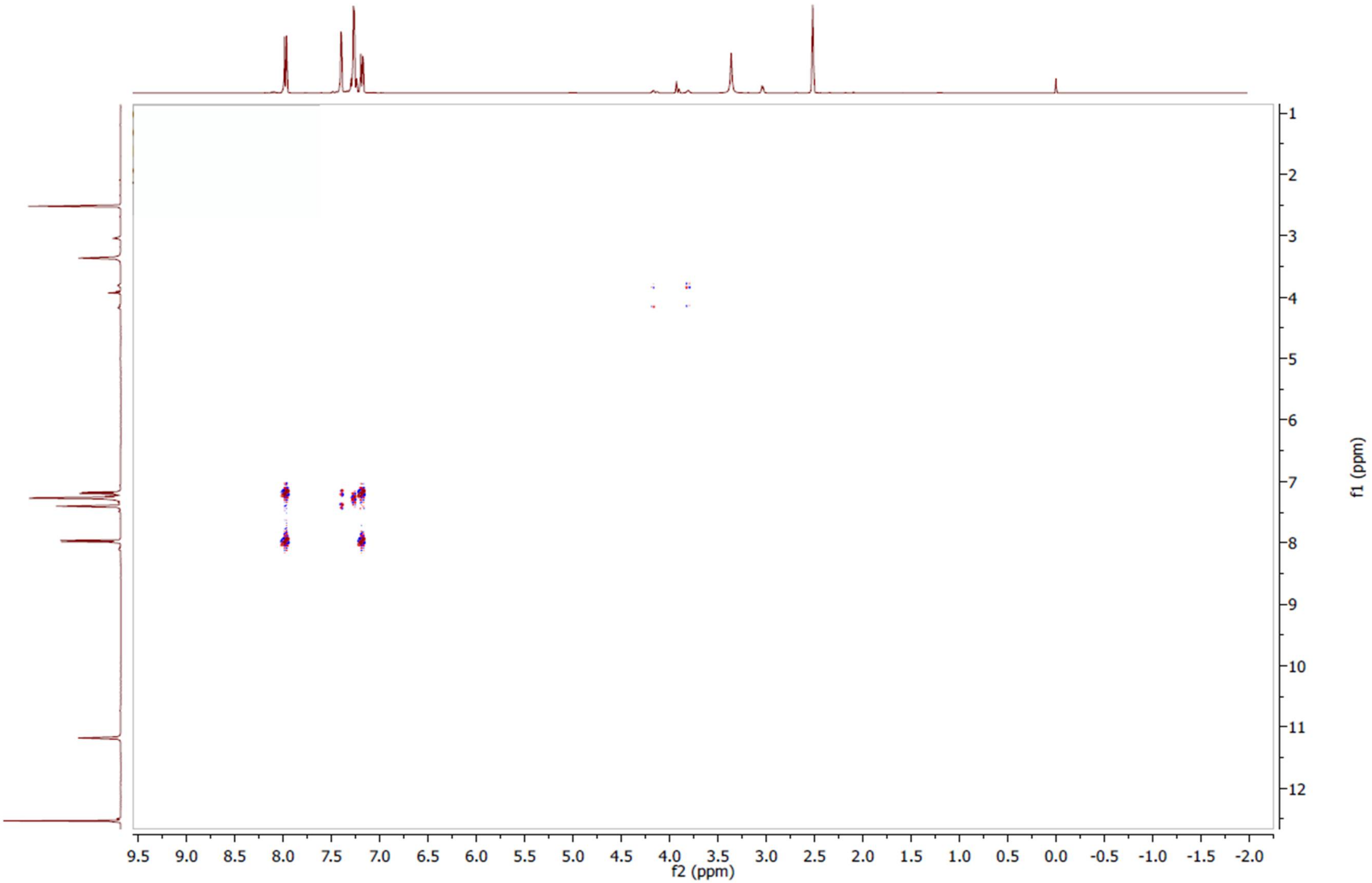

Page S20: Figure S18. COSY spectrum of muracatane B (**3**) (400 MHz, DMSO-d_6).

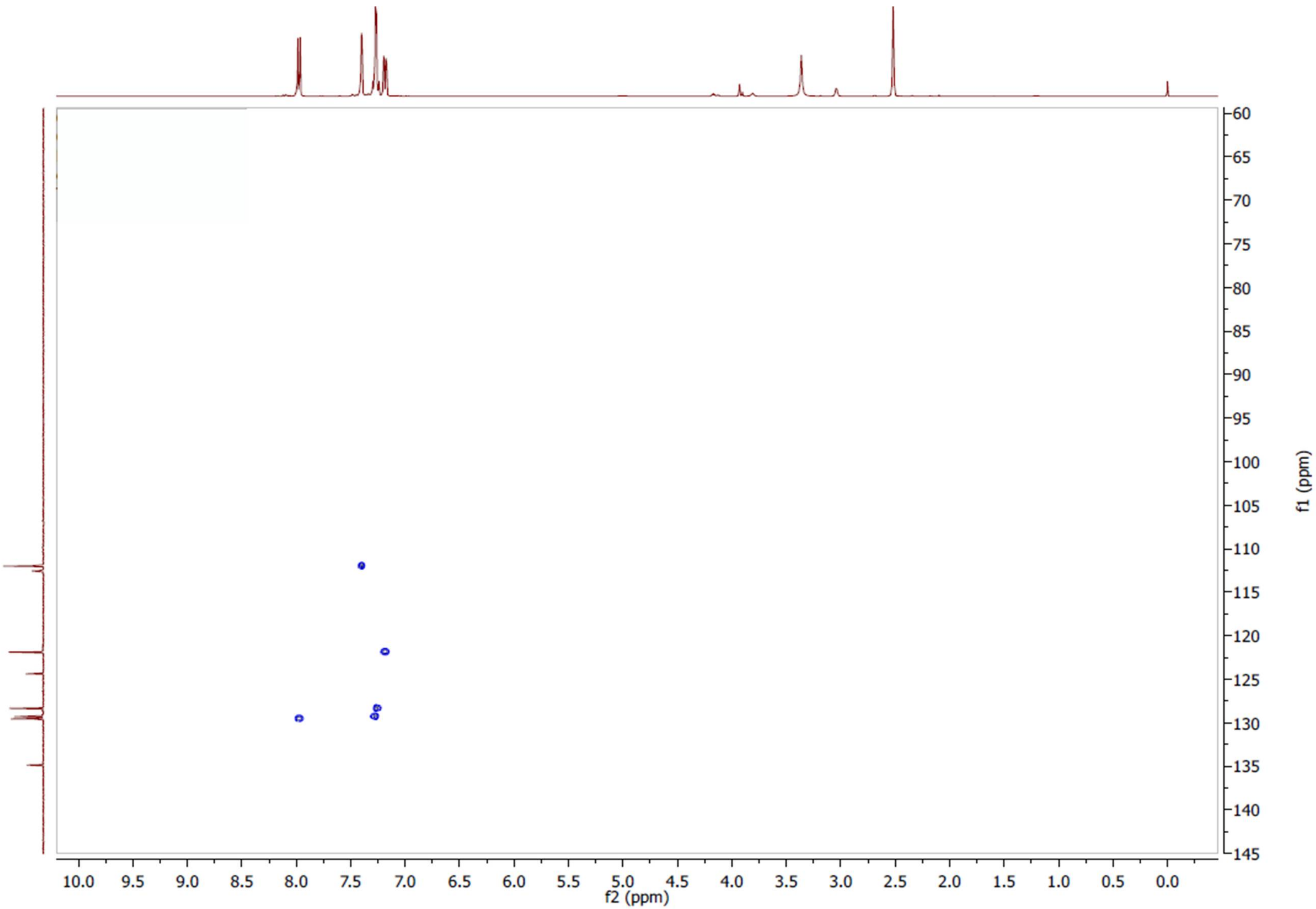

Page S21: Figure S19. HSQC spectrum of muracatane B (**3**) (400 MHz, DMSO-d_6).

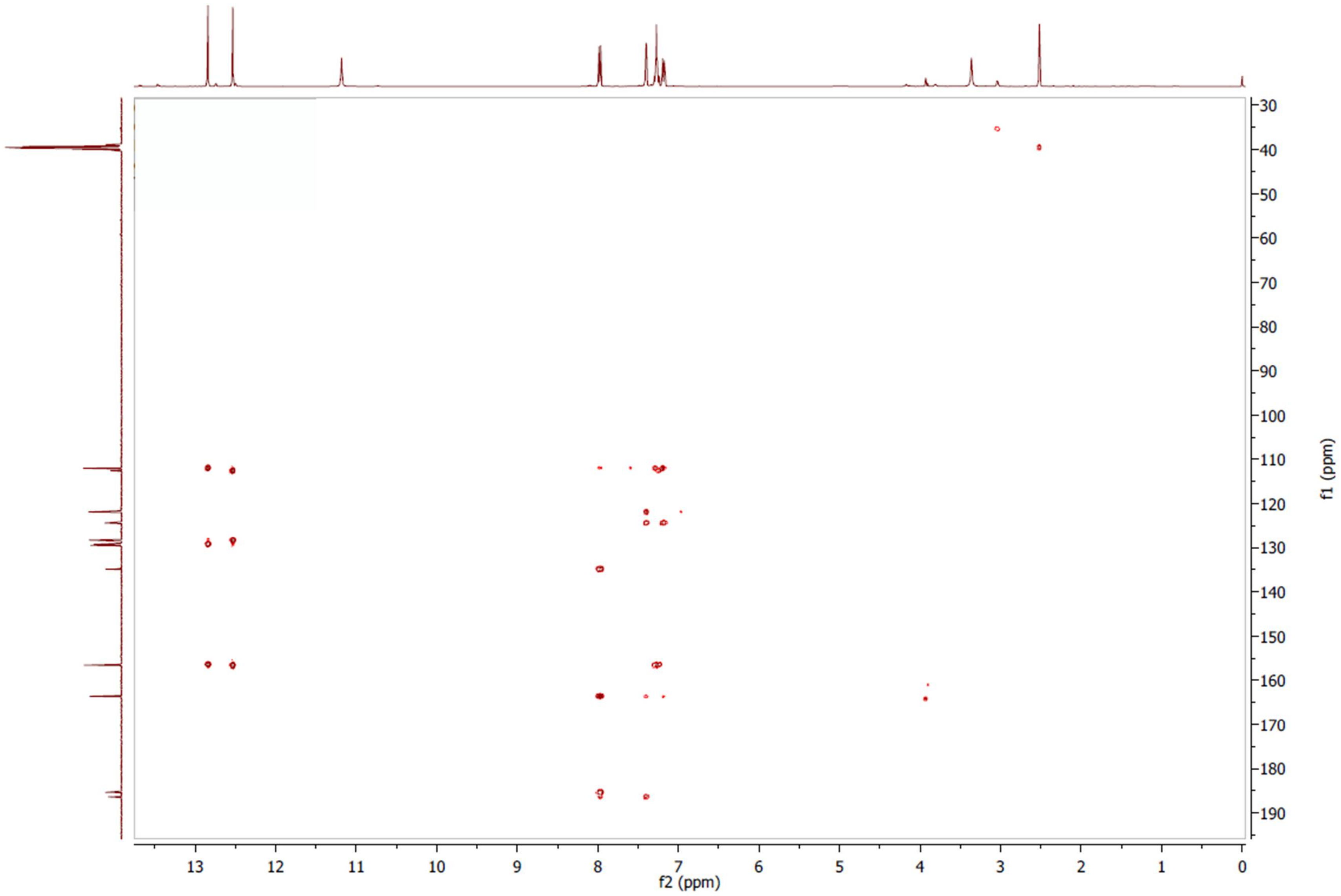

Page S22: Figure S20. HMBC spectrum of muracatane B (**3**) (400 MHz, DMSO-d$_6$).

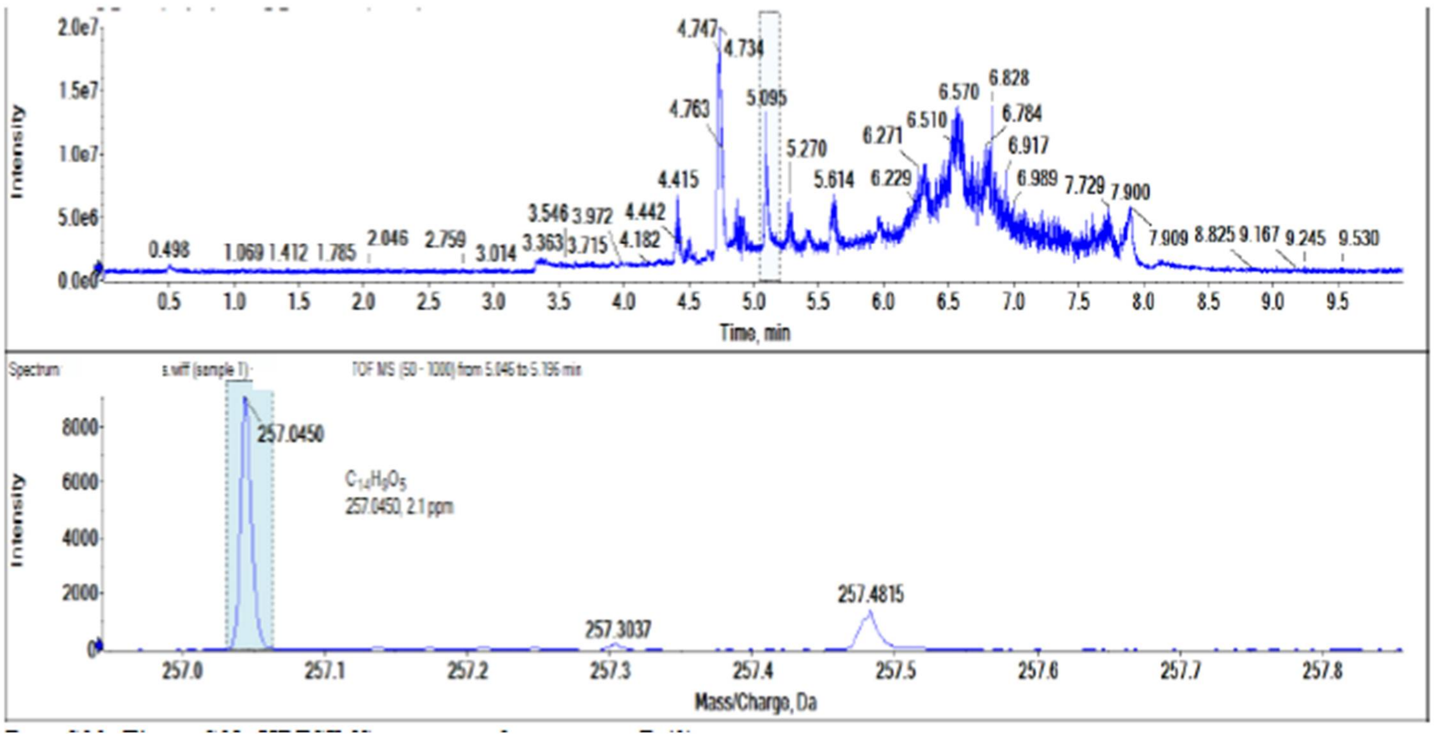

Page S23: Figure S21. HRESIMS spectrum of muracatane B (**3**).

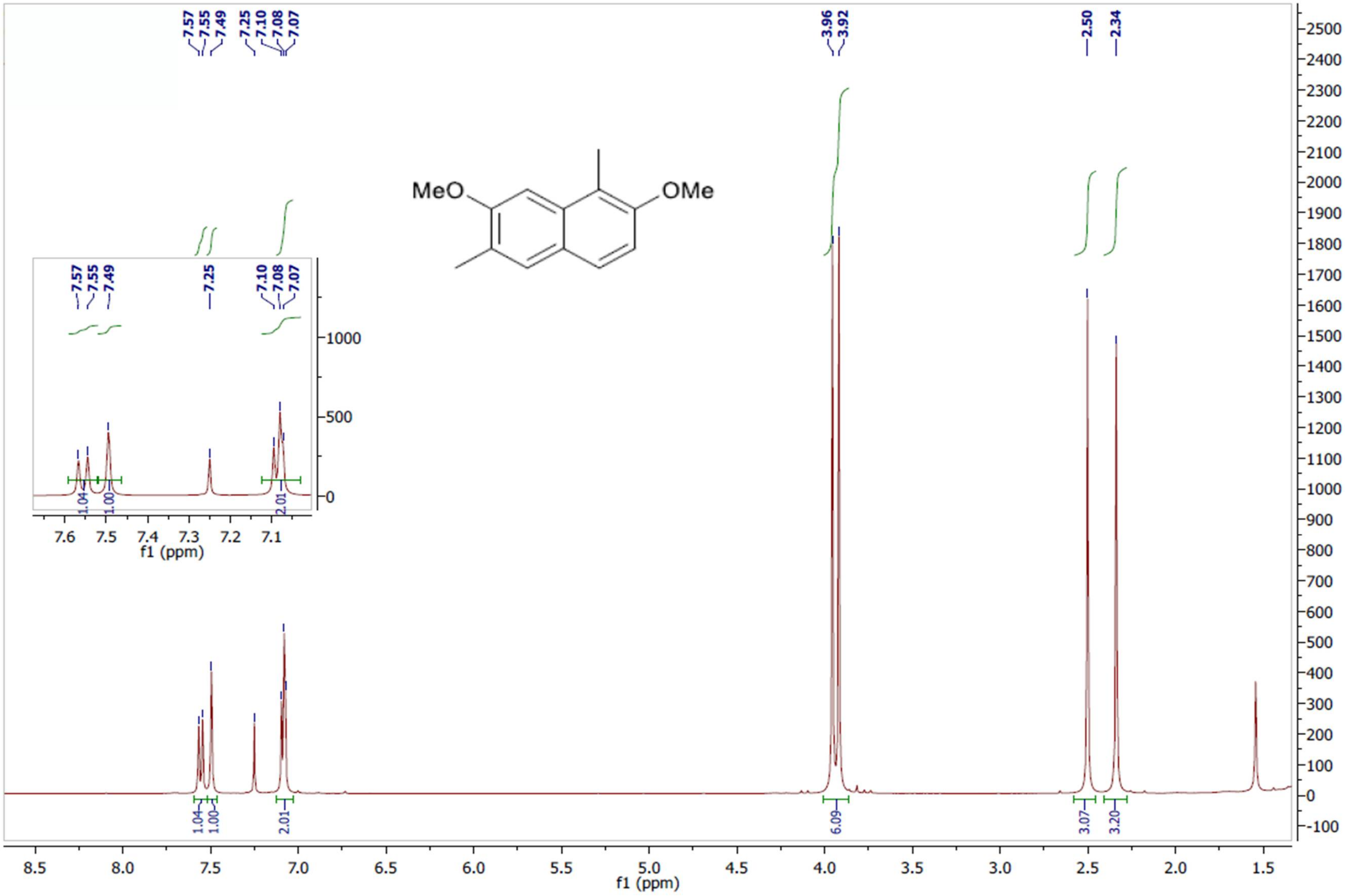

Page S24: Figure S22. ^{1}H NMR spectrum of muracatane C (**4**) (400 MHz, $CDCl_3$).

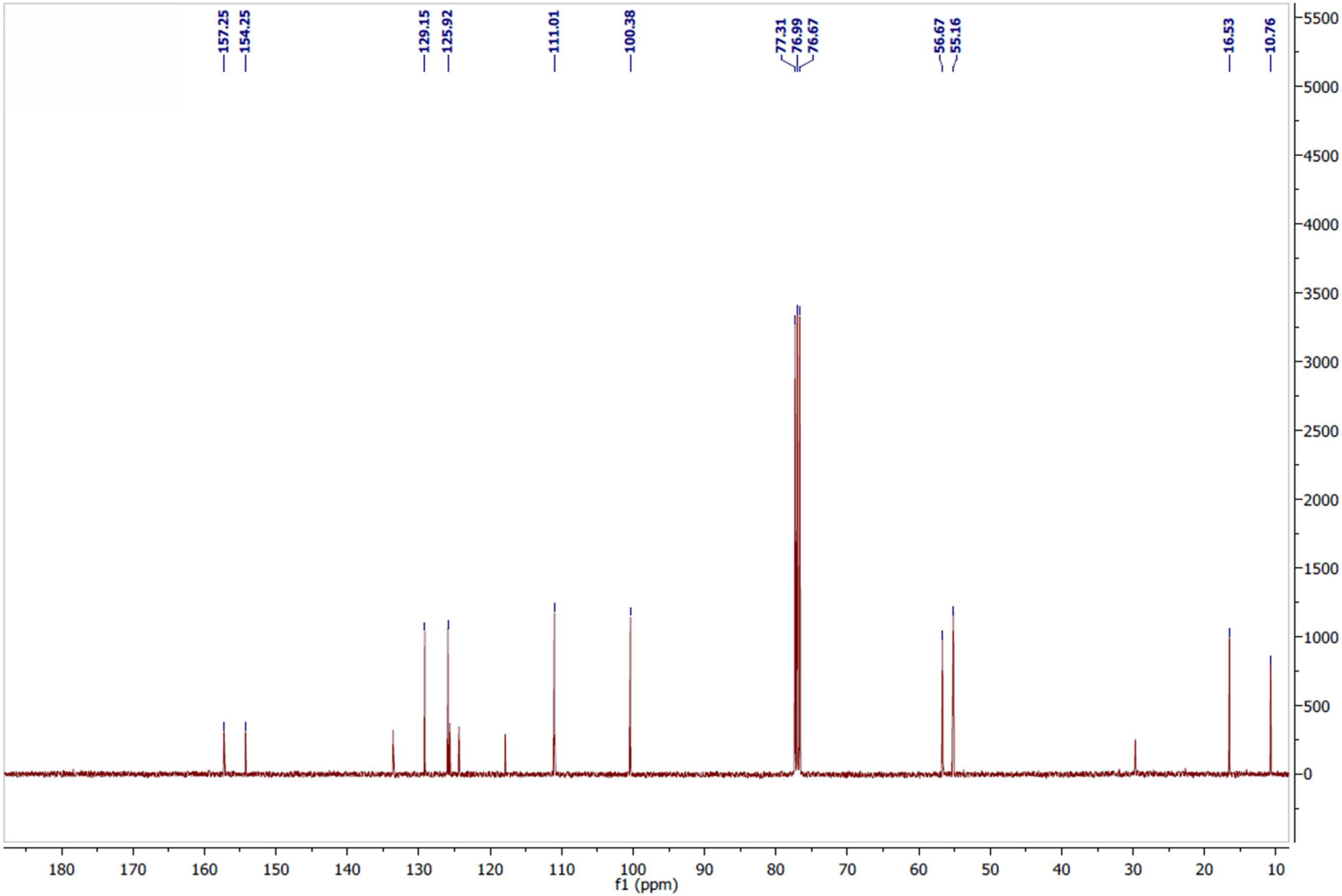

Page S25: Figure S23. ^{13}C NMR spectrum of muracatane C (**4**) (400 MHz, $CDCl_3$).

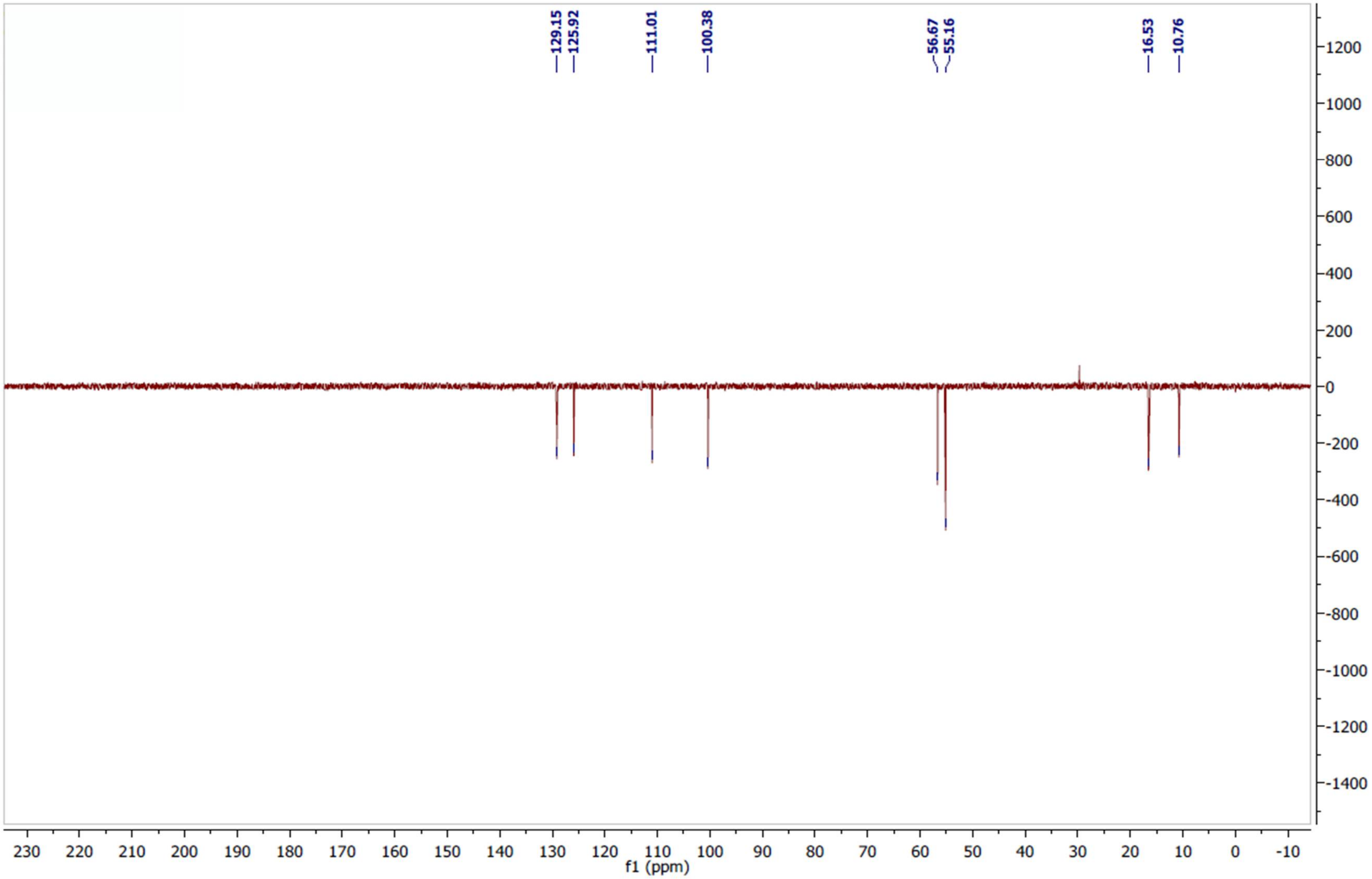

Page S26: Figure S24. DEPT spectrum of muracatane C (**4**) (400 MHz, $CDCl_3$).

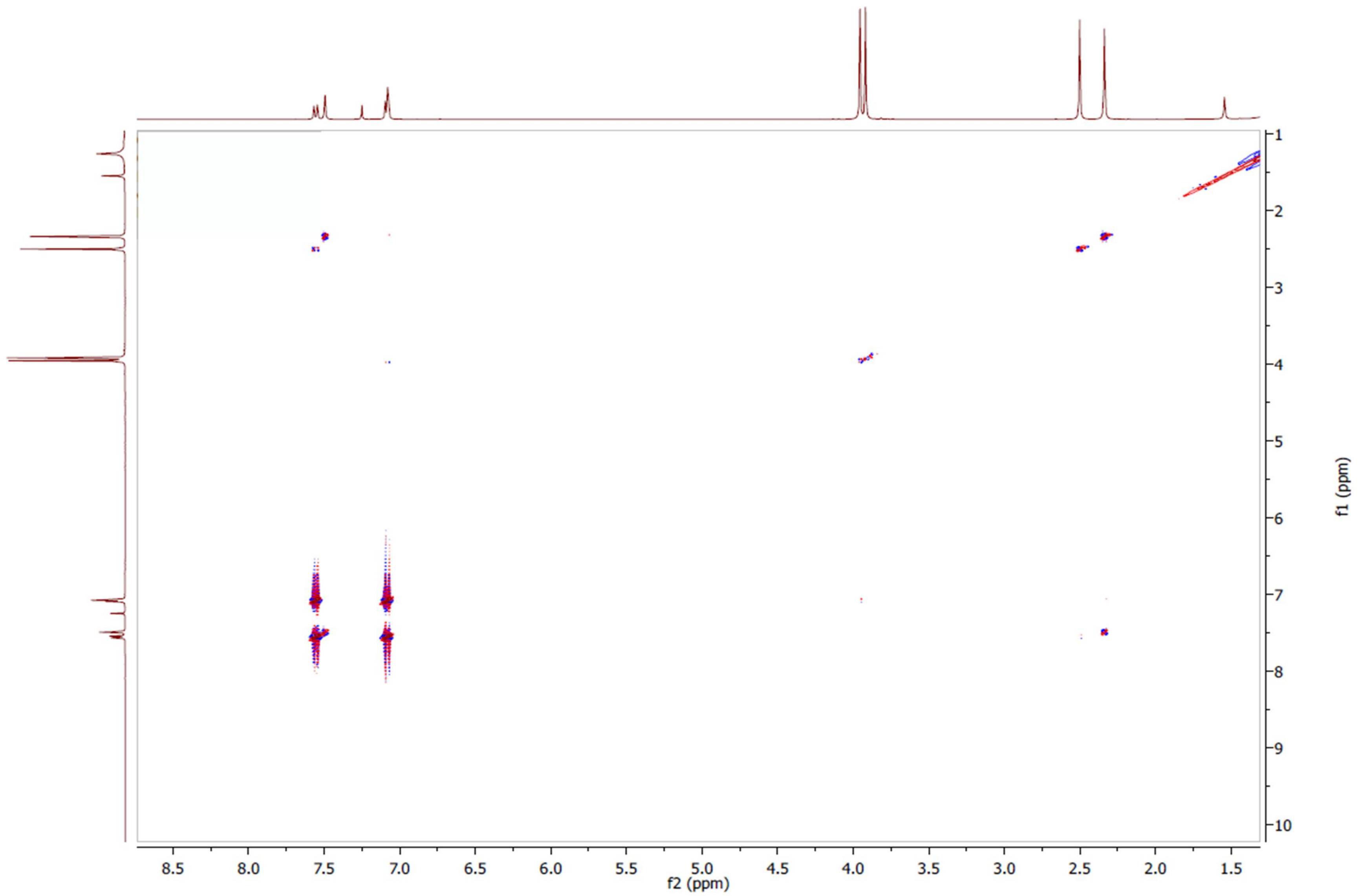

Page S27: Figure S25. COSY spectrum of muracatane C (**4**) (400 MHz, $CDCl_3$).

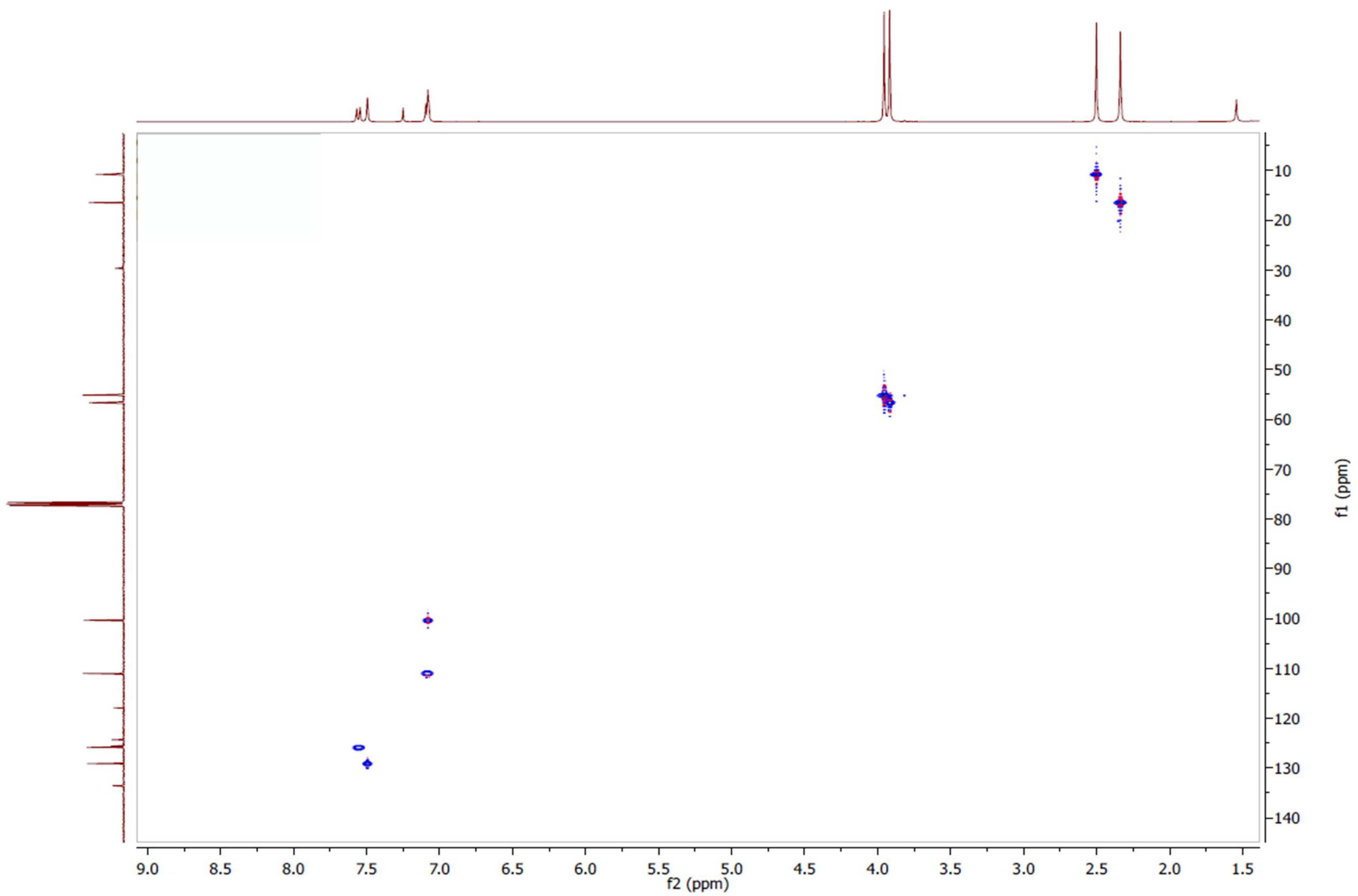

Page S28: Figure S26. HSQC spectrum of muracatane C (**4**) (400 MHz, $CDCl_3$).

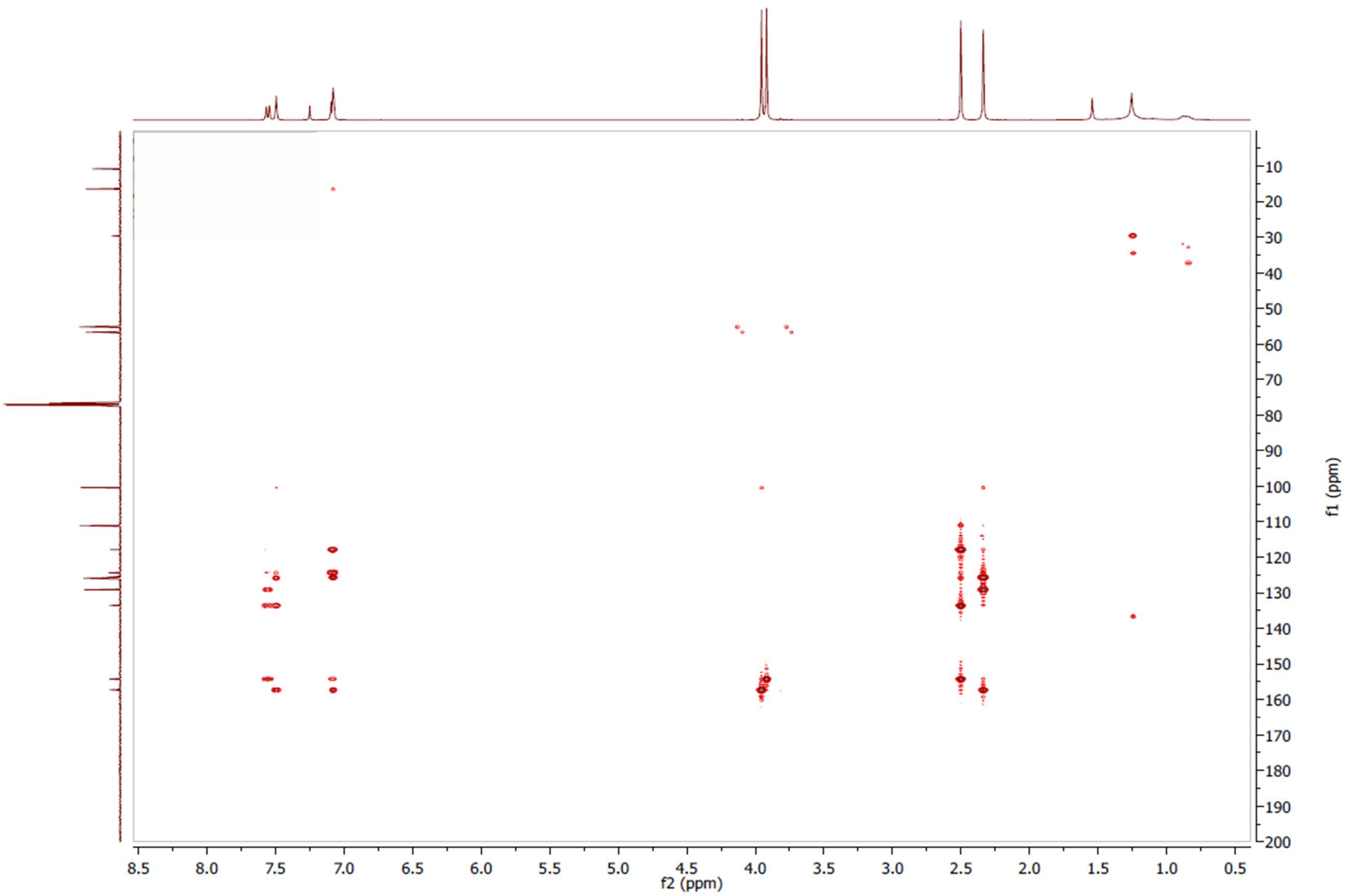

Page S29: Figure S27. HMBC spectrum of muracatane C (**4**) (400 MHz, $CDCl_3$).

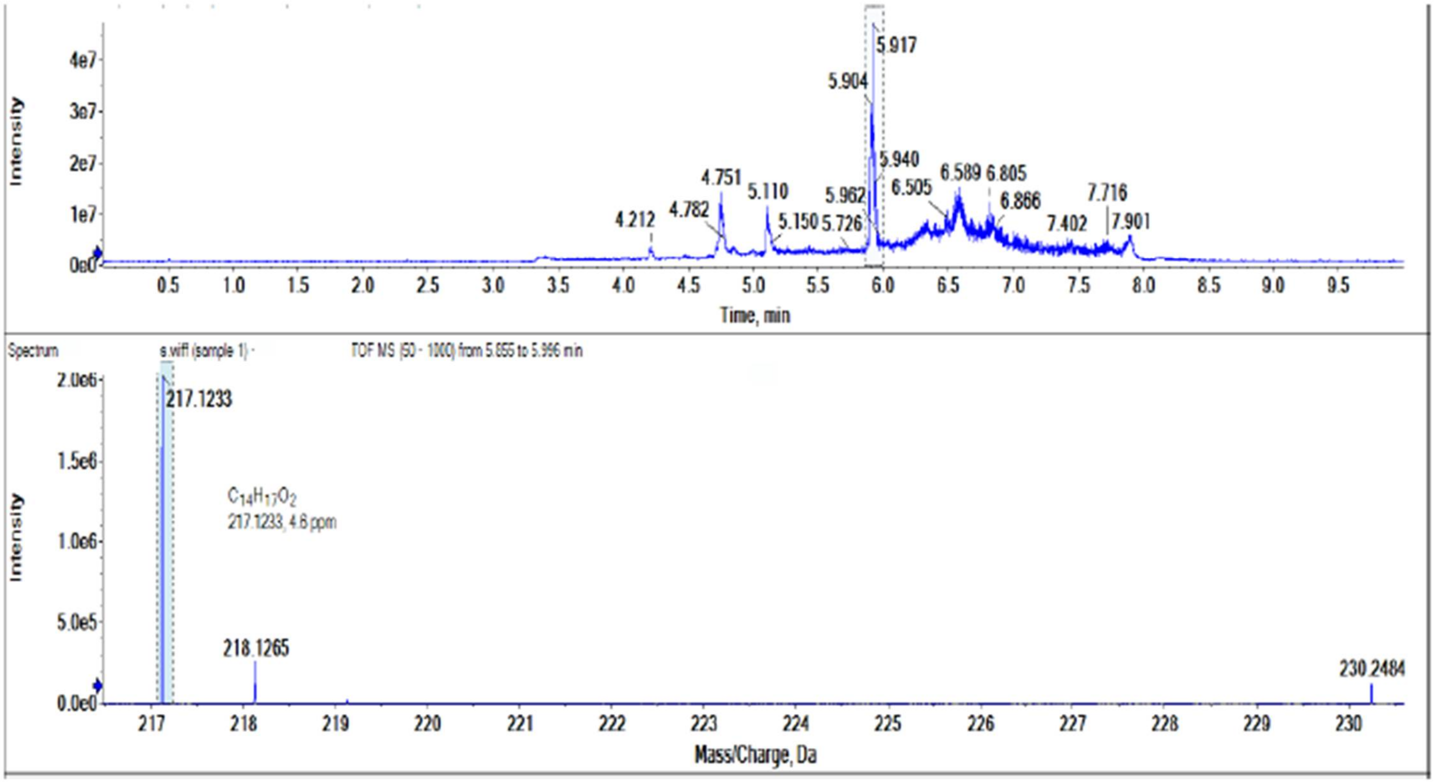

Page S30: Figure S28. HRESIMS spectrum of muracatane C (**4**).

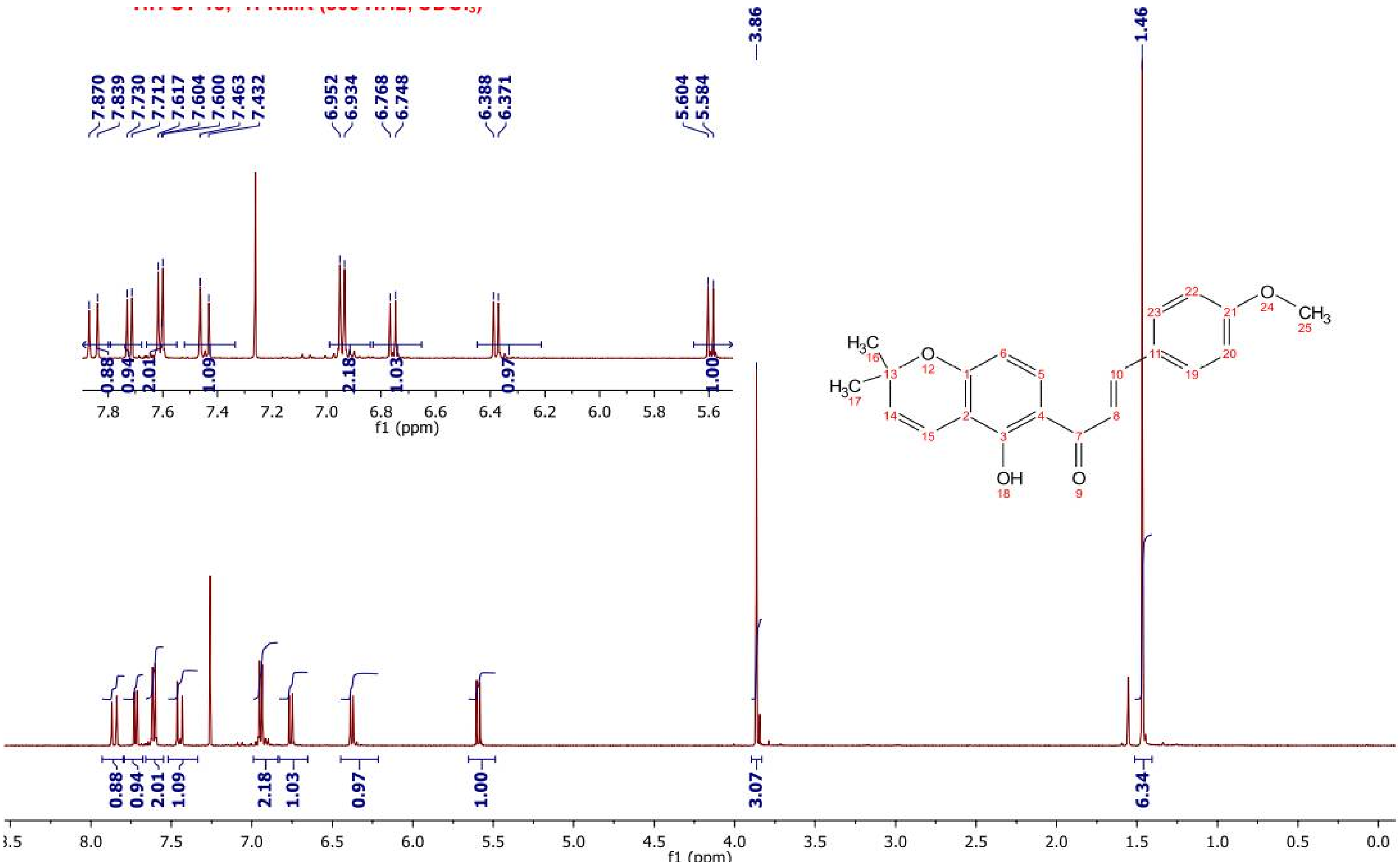

Page S31: Figure S29. ^{1}H NMR spectrum of 4-methoxylonchocarpin (**5**)
